# Comprehensive Characterization of Bihormonal Cells and Endocrine Cell Lineages in Mammalian Pancreatic Islets

**DOI:** 10.1002/advs.202416326

**Published:** 2025-05-29

**Authors:** Xin‐Xin Yu, Peng Peng, Yi‐Ning Wang, Mao‐Yang He, Shuang He, Chen‐Tao Jin, Liu Yang, Xi Wang, Jia‐Xi Zheng, Jie Gao, Cheng‐Ran Xu

**Affiliations:** ^1^ State Key Laboratory of Female Fertility Promotion Department of Medical Genetics School of Basic Medical Sciences Peking University Beijing 100191 China; ^2^ Peking‐Tsinghua Center for Life Sciences Peking University Beijing 100871 China; ^3^ PKU‐Tsinghua‐NIBS Graduate Program Peking University Beijing 100871 China; ^4^ School of Life Sciences Peking University Beijing 100871 China; ^5^ Department of Hepatobiliary Surgery Peking University People's Hospital Beijing 100044 China

**Keywords:** δ‐cell subpopulations, bihormonal cells, endocrine cell heterogeneity, gene coexpression network, imaging flow cytometry, pancreatic islets, single‐cell RNA sequencing

## Abstract

Understanding the role and prevalence of bihormonal cells in pancreatic islets and their potential in β‐cell restoration is critical but remains ambiguous. Using genetically engineered mouse strains with specific fluorescent markers and advanced imaging flow cytometry, it is found that bihormonal cells are exceedingly rare. Single‐cell RNA sequencing reveals that *Gcg* ^+^ *Ppy* ^+^ and *Gcg* ^+^ *Ins* ^+^ bihormonal cells closely resemble α‐cells or PP‐cells and α‐cells, respectively, indicating they are neither unique lineages nor transitional states. Dual‐recombinase lineage tracing further demonstrates that embryonic *Gcg^+^Ins^+^* cells resolve into monohormonal α‐cells. Applying these insights, the scarcity of bihormonal cells in diabetic mouse models is confirmed, suggesting a limited role in β‐cell regeneration. By excluding bihormonal influences, endocrine cell classification is redefined in mouse and human islets through gene coexpression network analysis, identifying distinct subtypes and regulatory modules while uncovering species‐specific differences. Additionally, two unique δ‐cell subpopulations are identified in human islets. Collectively, this study provides a comprehensive characterization of bihormonal cells, refines endocrine cell taxonomy, and underscores the translational challenges in modeling human islet biology in mice.

## Graphical abstract

This study integrates dual‐reporter genetics, imaging flow cytometry, and single‐cell sequencing to characterize rare bihormonal cells in mouse and human pancreatic islets. Gcg⁺Ins⁺ cells resemble α‐cells rather than transitional states. Cross‐species gene network analysis refines islet cell taxonomy and reveals human‐specific δ‐cell subtypes, advancing the understanding of endocrine identity and plasticity.

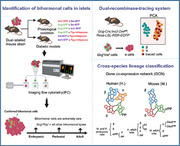

## 1 Introduction

The adult endocrine pancreas comprises specialized cells, namely α‐cells (GCG^+^), which secrete glucagon; β‐cells (INS^+^), which are responsible for insulin secretion; δ‐cells (SST^+^), which secrete somatostatin; and PP‐cells (PPY^+^), which produce pancreatic polypeptides. These distinct endocrine cell types are organized into pancreatic islets, which play a central role in maintaining glucose homeostasis. Ghrelin‐secreting ε‐cells (GHRL^+^), present predominantly during embryonic stages, serve as progenitors for both α‐ and PP‐cells but are largely absent in the adult mouse pancreas.^[^ ^1^ ^]^ Although endocrine cells typically specialize in producing a single hormone, the existence, frequency, and functional significance of bihormonal cells—those capable of secreting two hormones—remain active areas of investigation and discussion.

Bihormonal cells have been reported across multiple species, including zebrafish^[^ ^2^ ^]^ mice,^[^ ^3^ ^]^ rats,^[^ ^4^ ^]^ pigs,^[^ ^5^ ^]^ baboons,^[^ ^6^ ^]^ and humans.^[^ ^7^ ^]^ Immunostaining and imaging methods have revealed various coexpression patterns of endocrine hormones in mice.^[^ ^3^, ^8^ ^]^ Further research using lineage tracing and immunofluorescence has shown coexpression of PPY with other hormones in adult mouse islets.^[^ ^3^, ^9^ ^]^ Similar patterns of multihormonal cell expression have been observed in human embryonic pancreata, where cells were found to coexpress two or three hormone types.^[^ ^7^, ^10^ ^]^ Some studies have proposed that these bihormonal cells constitute unique cellular populations characterized by distinct gene expression profiles.^[^ ^9b^ ^]^ However, accurately identifying and quantifying these cells remains challenging due to limitations in immunostaining techniques, such as hormone diffusion, overlapping fluorescence signals, and variability in antibody sensitivity.

Single‐cell RNA sequencing (scRNA‐seq) has substantially advanced our understanding of bihormonal and multihormonal cells in both mice and humans.^[^ ^1^, ^11^ ^]^ For instance, a study using the Fluidigm C1 platform identified a human islet cell population with transcriptional features of both α‐ and β‐cells.^[^ ^11a^ ^]^ Using the 10× Genomics platform, van Gurp et al. also observed bihormonal cells in human islets, particularly those coexpressing INS and GCG,^[^ ^11b^ ^]^ and reported the presence of multihormonal cells in embryonic mouse pancreas.^[^ ^11c^ ^]^ However, accurately quantifying the proportion of such cells remains challenging. While one study using qPCR estimated that up to 46% of human islet cells may express two or more hormones,^[^ ^11d^ ^]^ another employing FACS followed by Smart‐seq2 scRNA‐seq reported much lower frequencies (0.9–1.8%).^[^ ^11e^ ^]^ These discrepancies underscore the technical limitations of current methodologies, particularly the influence of throughput and doublet contamination in droplet‐based systems.

Beyond their potential role in islet function, bihormonal cells have gained attention in the context of β‐cell regeneration and transdifferentiation. In juvenile mice, δ‐cell–dependent β‐cell regeneration has been observed following diphtheria toxin or STZ‐induced β‐cell ablation, suggesting potential avenues for diabetes recovery.^[^ ^12^ ^]^ Additionally, intermediate cells coexpressing the mesenchymal marker *vimentin* and the α‐cell marker *MafB* have been implicated in the regeneration of β‐like cells after STZ treatment.^[^ ^13^ ^]^ These findings suggest that non‐β endocrine cells may contribute to β‐cell replacement and highlight the plasticity of endocrine lineages. However, more recent studies have challenged the extent to which such transdifferentiation occurs following STZ‐induced injury.^[^ ^14^ ^]^ This raises the possibility that β‐cell regeneration may involve a transitional bihormonal cell state originating from endocrine progenitors or other islet cell types.

Given these conflicting observations, a clearer understanding of the prevalence, identity, and plasticity of bihormonal cells is essential. In this study, we leverage advanced dual‐reporter mouse models, imaging flow cytometry (IFC), and high‐resolution single‐cell transcriptomics to systematically characterize bihormonal cells across developmental stages and species, with the goal of elucidating their biological significance in both homeostasis and regeneration.

## 2 Results

### 2.1 Dual‐Fluorescent Labeling of Mouse Pancreatic Endocrine Cells

**Figure 1:**
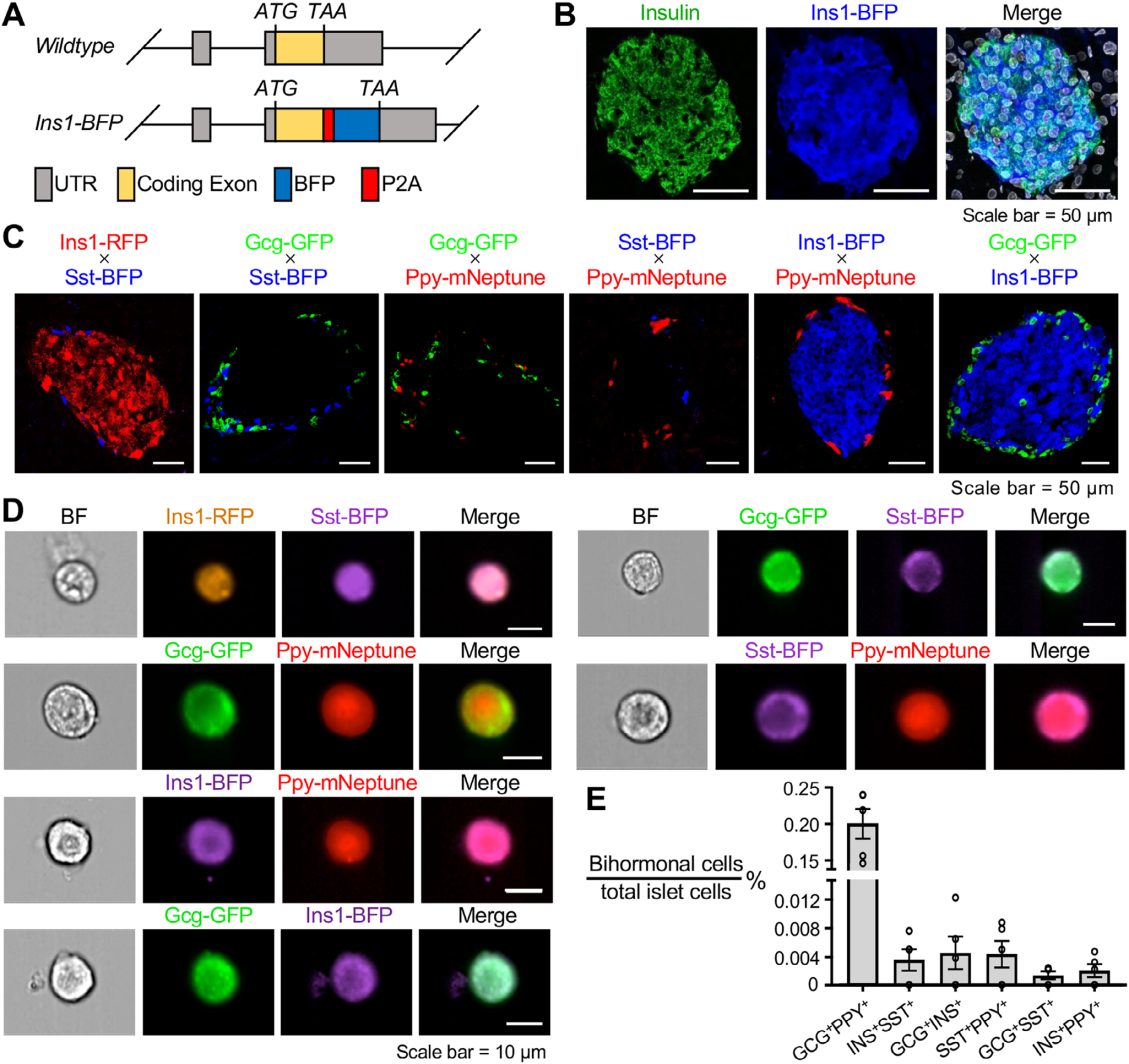
Genetic labeling and identification of bihormonal endocrine cells in adult mouse pancreatic islets. A) Schematic of the *Ins1‐P2A‐BFP* knock‐in construct, in which a *P2A* self‐cleaving peptide and blue fluorescent protein (BFP) sequence are inserted immediately upstream of the *Ins1* stop codon (TAA). B) Representative immunofluorescence image of pancreatic islet sections from a P60 *Ins1‐BFP* mouse, showing insulin immunostaining (green), BFP fluorescence (blue), and nuclear counterstaining with DAPI (white). The merged image demonstrates strong colocalization of insulin and BFP signals. Scale bars, 50 µm. C) Confocal images verifying dual‐fluorescent labeling in six genetically engineered mouse lines. Distinct color combinations correspond to specific dual‐labeled endocrine cell types in P60 islets. Scale bars, 50 µm. D) Representative IFC images showing individual dual‐positive cells from six dual‐labeled mouse strains, confirming the presence of bona fide bihormonal cells. Scale bars, 10 µm. E) Quantification of true dual‐positive endocrine cells as a percentage of total islet cells in P60 mouse pancreata. Data are presented as mean ± SEM (*n *= 5 mice per group).

To gain a comprehensive understanding of bihormonal cells in mouse pancreatic islets, we utilized genetically engineered mouse strains with fluorescent reporters specific to major endocrine lineages. These included *Gcg‐GFP* ^[^ ^11g^ ^]^ for α‐cells, *Sst‐BFP* ^[^ ^1a^ ^]^ for δ‐cells, *Ppy‐mNeptune* ^[^ ^1a^ ^]^ for PP‐cells, and a novel knock‐in strain, *Ins1‐BFP*, for β‐cells (**Figure** **1**A). The performance of the *Ins1‐BFP* strain was validated by immunofluorescence staining of adult mouse pancreatic tissue, which showed that 99.89% ± 0.05% of insulin‐positive cells were labeled with BFP (Figure 1B). To further confirm reporter specificity at the single‐cell level, we sorted BFP⁺ and BFP⁻ cells and performed immunostaining using antibodies against insulin and glucagon. Nearly all insulin⁺ cells were found in the BFP⁺ population, while very few were present in BFP⁻ cells, confirming the high specificity of the *Ins1‐BFP* reporter for insulin‐expressing β‐cells (Figure S1A, Supporting Information). To avoid fluorescence interference between the *Ins1‐BFP* and *Sst‐BFP* strains, we introduced the transgenic *Ins1‐RFP* ^[^ ^15^ ^]^ strain as an alternative for β‐cell labelling.

Following the establishment of these base strains, we interbred them to generate six dual‐labeled mouse variants encompassing all possible pairwise combinations of endocrine lineages: *Ins1‐RFP; Sst‐BFP*, *Gcg‐GFP; Sst‐BFP*, *Gcg‐GFP; Ppy‐mNeptune*, *Sst‐BFP; Ppy‐mNeptune*, *Ins1‐BFP; Ppy‐mNeptune*, and *Gcg‐GFP; Ins1‐BFP*. To verify the accuracy of our dual‐fluorescence labeling strategy, we performed cryosectioning and imaging on pancreatic samples from at least three adult mice per variant. For each mouse, a minimum of 10 intact islets were imaged to ensure representative coverage of reporter expression. Across all variants, endogenous fluorescent signals were consistently detected within the islets (Figure 1C; Figure S1B, Supporting Information), confirming the robustness of our dual‐labeling system for identifying hormone‐producing endocrine cells in the mouse pancreas.

### 2.2 Identification of Bihormonal Cells in Adult Mouse Islets by Imaging Flow Cytometry

To quantify the prevalence of bihormonal cells, we harvested adult mouse islets and enzymatically dissociated them into single‐cell suspensions. The digestion process was optimized to minimize multicellular aggregation due to under‐digestion while preventing cell damage from over‐digestion.^[^ ^16^ ^]^ Based on cell viability assessments, a five‐minute digestion time was determined to be optimal (Figure S1C, Supporting Information). Using FACS, we analyzed six dual‐fluorescent cell populations isolated from the respective dual‐reporter mouse lines. Each dual‐positive populations accounted for ≈0.2% to 0.5% of the total islet cell population (Figure S1D, Supporting Information). However, subsequent microscopic examination revealed that a large proportion of these dual‐positive events were in fact cellular doublets or small clusters, rather than bona fide bihormonal cells (Figure S1E, Supporting Information).

To overcome the inherent limitations of FACS combined with microscopy, such as potential loss of rare cells during centrifugation, nonsystematic cell loss during sample processing, and challenges in quantifying fluorescence intensity, we employed IFC. This innovative technique combines the high‐throughput capabilities of flow cytometry with the detailed imaging acuity of microscopy.^[^ ^17^ ^]^ For each dual‐labeled combination, over 100 000 cytometric events were acquired from at least five biological replicates (Figure 1D; Figure S1F, Supporting Information), enabling robust quantification of true bihormonal cells.

Our IFC analysis confirmed that genuine bihormonal cells are extremely rare. Among all populations, Gcg‐GFP^+^Ppy‐mNeptune^+^ (GCG^+^PPY^+^) cells were the most abundant, representing ≈0.2% of total islet cells, while other bihormonal combinations accounted for only 0.001% to 0.005% (Figure 1E). Notably, most authentic bihormonal cells were found within dual‐fluorescent gates, which also frequently contained doublets or triplets (Figure S1G, Supporting Information). Overlapping hormone signals at the contact points between doublets may lead to misidentification of bihormonal cells by conventional imaging techniques (Figure S1G, Supporting Information). However, as doublets constituted only ≈2% of the total population, their impact on overall quantification was minimal.

In conclusion, our results provide direct evidence for the existence of bihormonal cells in adult mouse pancreatic islets, although they represent only a very small fraction of the total endocrine cell population.

### 2.3 Temporal Dynamics of Bihormonal Cell Emergence during Pancreatic Development

**Figure 2:**
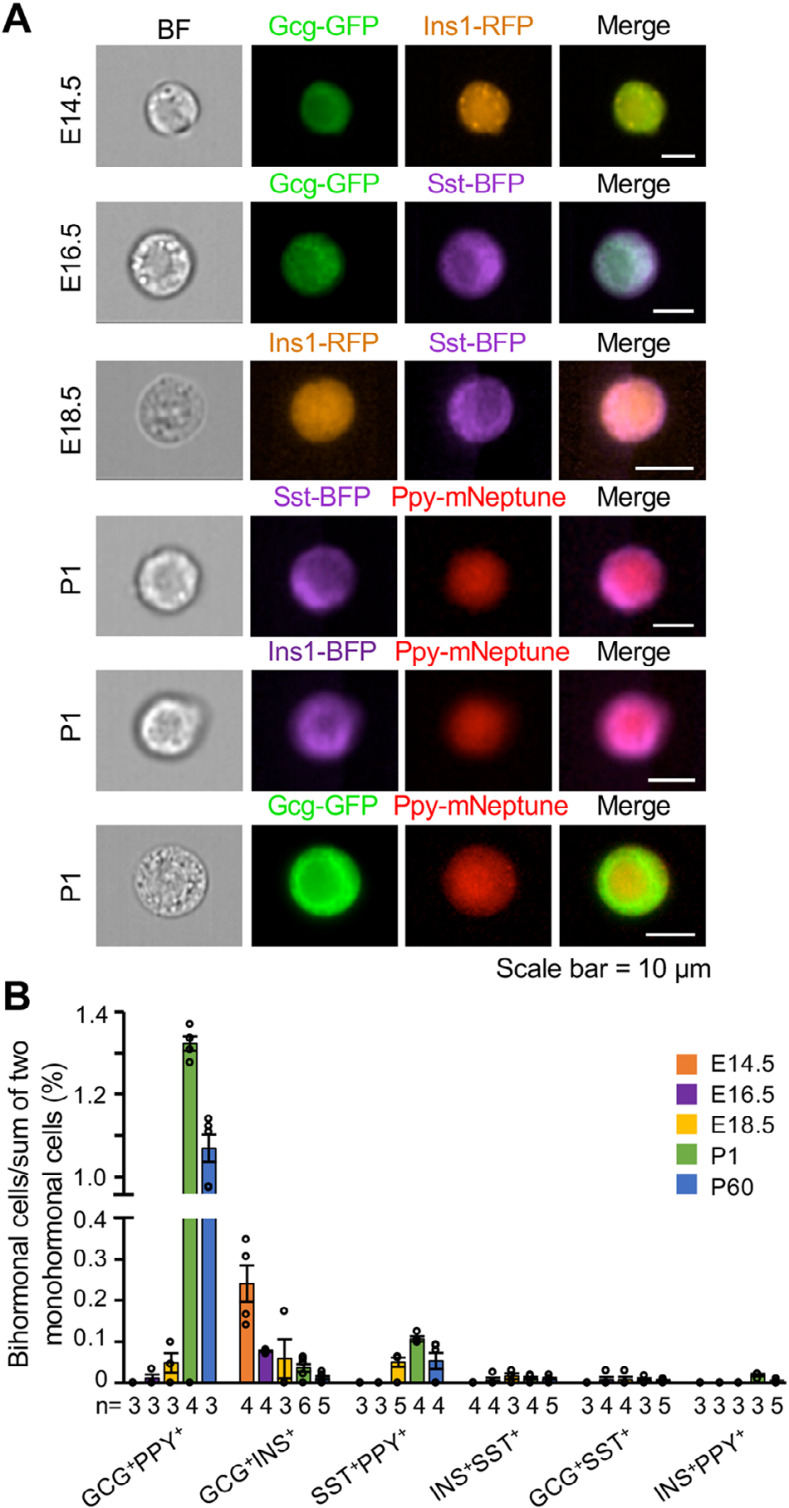
Developmental dynamics of bihormonal cells in the mouse pancreas. A) Representative IFC images of bihormonal cells at embryonic stages (E14.5, E16.5, and E18.5) and postnatal day P1. Brightfield (BF) and corresponding fluorescent channels are shown for different hormone combinations. Scale bars, 10 µm. B) Quantification of six types of bihormonal cells expressed as a percentage of their corresponding monohormonal cell populations at each developmental stage. Data are presented as mean ± SEM. The number of mice analyzed is indicated as n.

Utilizing our robust dual‐fluorescent mouse models in combination with live‐cell IFC, we systematically examined the temporal emergence of bihormonal cells throughout pancreatic development, focusing particularly on embryonic and perinatal stages (**Figure** **2**A).

Among the bihormonal populations, Gcg‐GFP^+^Ppy‐mNeptune^+^ cells constituted ≈1.3% of the combined Gcg‐GFP⁺ and Ppy‐mNeptune⁺ cell population during the perinatal stage, with this proportion decreasing slightly by postnatal day (P) 60 (Figure 2B). Gcg‐GFP^+^Ins1‐RFP^+^ cells peaked at ≈0.2% around embryonic day (E) 14.5 and gradually declined thereafter, reaching only ≈0.01% in the adult pancreas (Figure 2B). Consistent with the relatively late development of δ‐ and PP‐cells,^[^ ^1a^ ^]^ the frequency of Sst‐BFP^+^Ppy‐mNeptune^+^ cells peaked during the perinatal period (Figure 2B).

In contrast, three other bihormonal combinations, Ins1‐RFP^+^Sst‐BFP^+^, Gcg‐GFP^+^Sst‐BFP^+^, and Ins1‐BFP^+^Ppy‐mNeptune^+^, remained extremely rare across all developmental stages examined (Figure 2B).

Altogether, our analysis delineates the distinct temporal dynamics of various bihormonal cell types, highlighting their patterns of emergence and resolution throughout pancreas development.

### 2.4 Transcriptomic Analysis of Bihormonal Cells

Statistical analysis revealed that *Gcg^+^Ppy^+^* bihormonal cells consistently represented the most prevalent bihormonal population in mouse islets, surpassing other bihormonal cell types from the perinatal stage through adulthood (Figure 2B). To investigate the transcriptomic features of these cells, we isolated Gcg‐GFP^+^Ppy‐mNeptune^+^ cells from P60 mouse islets, along with monohormonal Gcg‐GFP*^+^* and Ppy‐mNeptune*^+^* cells as controls (Figure S2A, Supporting Information). Each cell was individually collected via mouth pipetting under microscopic guidance. In addition, we generated artificial doublets by manually combining one Gcg‐GFP^+^ cell with one Ppy‐mNeptune^+^ cell in the same tube. All collected cells were then subjected to scRNA‐seq using the Smart‐seq3xpress protocol (Smart‐seq3)^[^ ^18^ ^]^ (Figure S2B, Table S1, Supporting Information).

**Figure 3:**
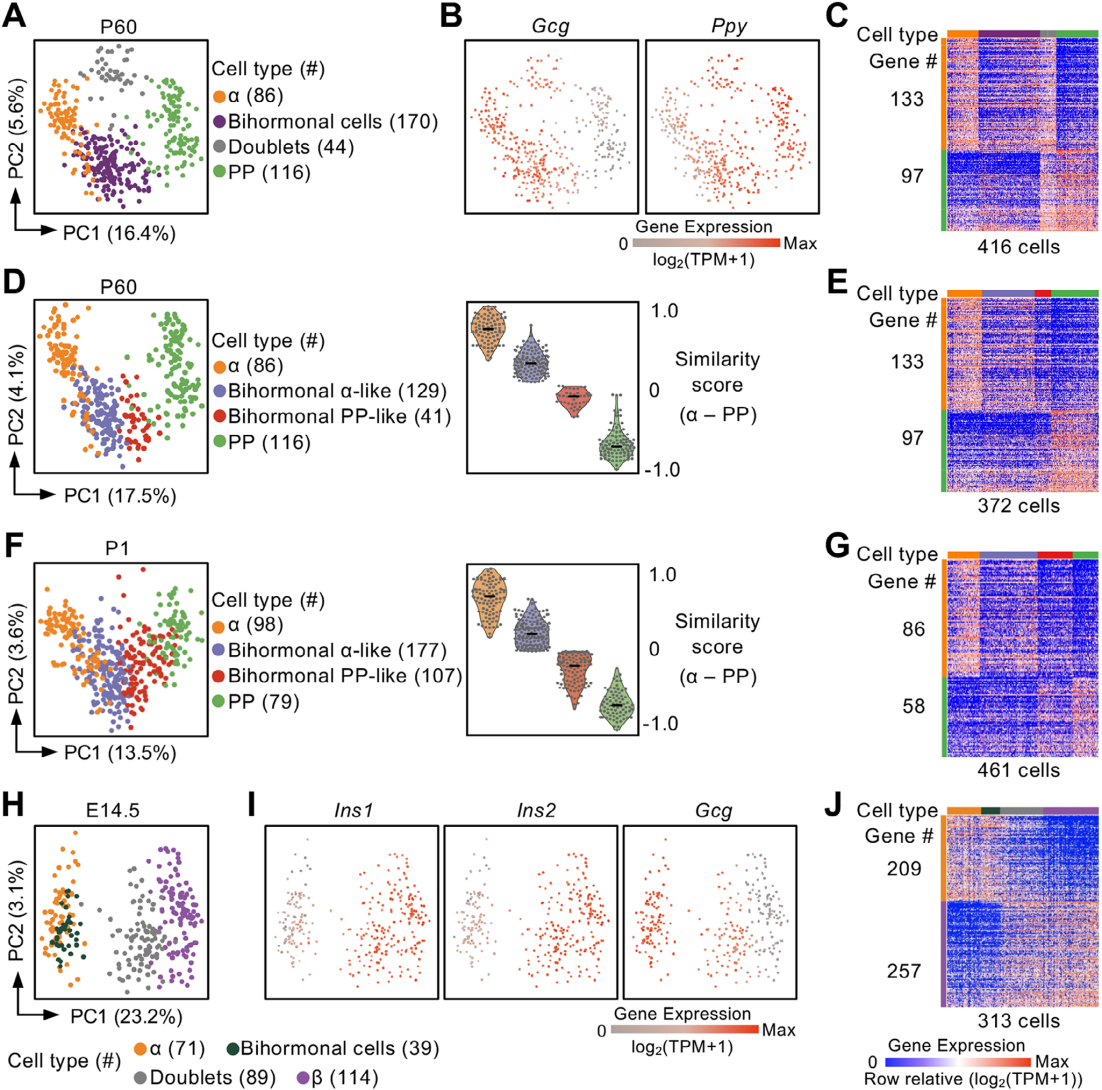
ScRNA‐seq analysis of bihormonal and monohormonal endocrine cells in the mouse pancreas. A) Principal component analysis (PCA) of scRNA‐seq data from P60 mouse islets, showing distinct clusters of α‐cells, *Gcg^+^Ppy^+^* bihormonal cells, doublets, and PP‐cells. The percentage of variance explained by each principal component (PC) is indicated. Cell counts are shown in brackets. B) PCA plots showing the expression levels of *Gcg* and *Ppy* in the cell types identified in (A). Each dot represents a single cell. C) Heatmap of differentially expressed genes (DEGs) between α‐ and PP‐cells from P60 mouse islets. Each column represents a single cell; each row represents a gene. The color coding of cell types follows that in (A). DEGs were identified using the Wilcoxon rank‐sum test (adjusted *p *≤ 0.05, log₂ fold change ≥ 0.5, pct.1 ≥ 0.5, pct.2 ≤ 0.9). D) PCA plot (left) comparing P60 α‐cells, bihormonal α‐like cells, bihormonal PP‐like cells, and PP‐cells. Violin plots (right) similarity scores to α‐ and PP‐cells. Black lines indicate the median values. E) Heatmap of DEGs between α‐ and PP‐cells as shown in (D), using the same cell color scheme. DEGs were identified using the Wilcoxon rank‐sum test (adjusted *p *≤ 0.05, log₂ fold change ≥ 0.5, pct.1 ≥ 0.5, pct.2 ≤ 0.9). F) PCA plot (left) comparing P1 α‐cells, bihormonal α‐like cells, bihormonal PP‐like cells, and PP‐cells. Violin plots (right) show similarity scores to α‐ and PP‐cells. Black lines indicate the median values. G) Heatmap of DEGs between α‐ and PP‐cells in P1 mouse islets, as shown in (F). DEGs were identified using the Wilcoxon rank‐sum test (adjusted *P *≤ 0.05, log₂ fold change ≥ 0.5, pct.1 ≥ 0.5, pct.2 ≤ 0.8). H) PCA plot of scRNA‐seq data from E14.5 mouse pancreas, showing β‐cells, α‐cells, and *Gcg^+^Ins^+^* bihormonal cells and doublets. Cell counts are indicated in brackets. I) PCA plots showing the expression of key marker genes in the cell types identified in (H). J) Heatmap of DEGs between α‐ and β‐cells from E14.5 mouse pancreata, corresponding to the groups in (H). DEGs were identified using the Wilcoxon rank‐sum test (adjusted *P *≤ 0.05, log₂ fold change ≥ 0.5, pct.1 ≥ 0.5, pct.2 ≤ 0.8).

Principal component analysis (PCA) of the P60 single‐cell transcriptomic data revealed a clear separation between artificial doublets and genuine *Gcg^+^Ppy^+^* bihormonal cells, although both clusters were positioned between the α‐ and PP‐cell clusters (**Figure** **3**A,B). Artificial doublets co‐expressed differentially expressed genes (DEGs) characteristic of both α‐ and PP‐cells, whereas bona fide bihormonal cells exhibited distinct and consistent transcriptional profiles (Figure 3C, Table S2, Supporting Information).

Similarity score analysis further demonstrated that GFP⁺mNeptune⁺ cells clustered predominantly with either α‐ or PP‐cells, allowing classification of these bihormonal cells into two subgroups: α‐like and PP‐like *Gcg^+^Ppy^+^* cells (Figure 3D; Figure S2C, Supporting Information). Each subgroup was marked by high expression of α‐cell or PP‐cell signature genes, respectively (Figure 3D,E). Gene coexpression network (GCN) analysis, a robust method for identifying cell subtypes, revealed that α‐like and PP‐like *Gcg^+^Ppy^+^* cells shared co‐regulated gene modules with their respective monohormonal counterparts (Figure S2D, Supporting Information). Remarkably, these α‐like and PP‐like *Gcg* ^+^ *Ppy* ^+^ cells were identifiable as early as at the P1 stage (Figure 3F,G; Figure S2E–G, Tables S1,S2, Supporting Information). Expression analysis revealed a gradual decrease in *Gcg* expression and a corresponding increase in *Ppy* expression across the α‐cell → α‐like → PP‐like → PP‐cell axis (Figure S2H,I, Supporting Information). IFC analysis of P60 pancreas further validated these trends: GFP⁺mNeptune⁺ cells exhibited intermediate GFP and mNeptune intensities compared to α‐ and PP‐cells (Figure S2J, Supporting Information). Intriguingly, DEG analysis revealed no genes uniquely expressed in either P1 or P60 *Gcg^+^Ppy^+^* bihormonal cells (Figure S2K,L, Table S2, Supporting Information), indicating that these cells do not constitute a distinct lineage with unique transcriptional identity.

In addition, our data revealed the presence of *Gcg^+^Ins^+^* bihormonal cells during the embryonic stage (Figure 2B). We isolated Gcg‐GFP⁺Ins1‐RFP⁺ cells from E14.5 embryos along with monohormonal controls and subjected them to Smart‐seq3 scRNA‐seq (Figure S2B,M, Table S1, Supporting Information). Artificial doublets were also generated by manually combining a Gcg‐GFP^+^ cell with an Ins1‐RFP^+^ cell. PCA revealed that *Gcg^+^Ins^+^* cells clustered closely with α‐cells, whereas artificial doublets were positioned between the α‐ and β‐cell clusters (Figure 3H–J, Table S2, Supporting Information). A DEG heatmap further showed that *Gcg* ^+^ *Ins* ^+^ cells shared transcriptional features with α‐cells and did not exhibit unique gene expression signatures (Figure S2N, Table S2, Supporting Information). Moreover, *Ins* expression in *Gcg^+^Ins^+^* cells was markedly lower than in β‐cells, whereas *Gcg* expression was comparable to that in α‐cells (Figure 3I; Figure S2O, Supporting Information).

Taken together, these findings indicate that certain bihormonal cells exhibit transcriptomic features characteristic of their monohormonal counterparts, challenging the notion that these cells constitute distinct cellular types.

### 2.5 Dual‐Genetic Tracing Elucidates the Terminal Differentiation of *Gcg^+^Ins^+^* Cells

To determine the developmental fate of Gcg‐GFP*^+^* Ins1‐RFP^+^ bihormonal cells, we employed a dual‐genetic tracing approach integrating the Cre‐loxP and Dre‐rox recombination systems.^[^ ^19^ ^]^ We developed a novel reporter mouse strain, *Rosa‐loxP‐stop‐loxP‐rox‐stop‐rox‐EGFP* (*Rosa‐LSL‐RSR‐EGFP*), which enables EGFP expression only upon coactivation of both recombinases at the *Rosa26* locus (Figure S3A, Supporting Information).^[^ ^19^, ^20^ ^]^

To validate this system, *Rosa‐LSL‐RSR‐EGFP* mice were crossed with *Pdx1‐Cre^ER^* and *Ins2‐Dre^ER^* strains to specifically label β‐cells (Figure S3A, Supporting Information). Immunofluorescence assays showed that EGFP expression was absent in all control groups, including tamoxifen (TM)‐treated *Pdx1‐Cre^ER^; Rosa‐LSL‐RSR‐EGFP* and *Ins2‐Dre^ER^; Rosa‐LSL‐RSR‐EGFP* mice, as well as untreated *Pdx1‐Cre^ER^; Ins2‐Dre^ER^; Rosa‐LSL‐RSR‐EGFP* mice (Figure S3B, Supporting Information). INS^+^EGFP^+^ cells appeared only in TM‐treated *Pdx1‐Cre^ER^; Ins2‐Dre^ER^; Rosa‐LSL‐RSR‐EGFP* mice (Figure S3B, Supporting Information), confirming the system's specificity and efficiency.

**Figure 4:**
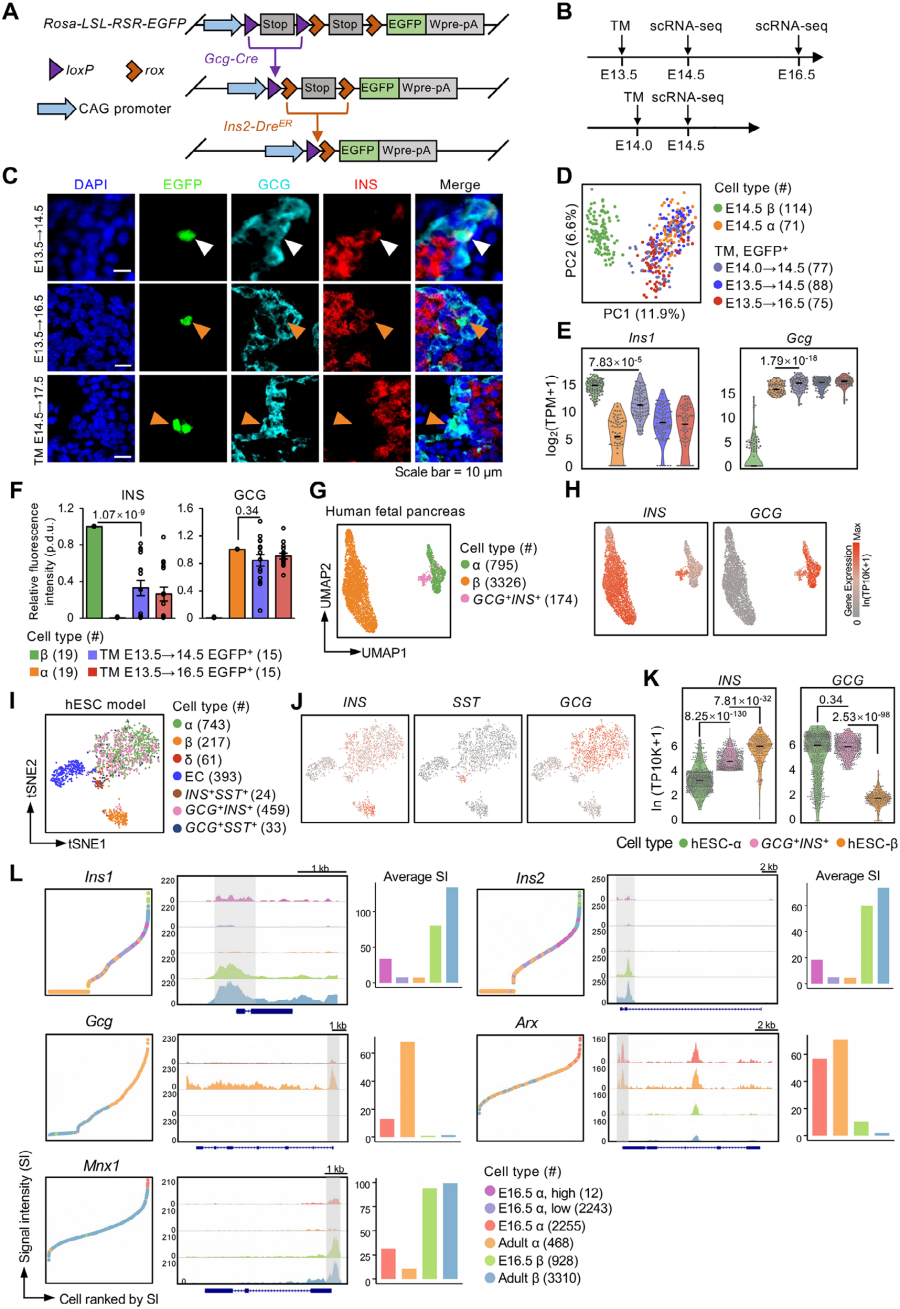
Dual‐recombinase‐mediated genetic tracing of *Gcg^+^Ins^+^* bihormonal cells during pancreatic development. A) Schematic of the *Gcg‐Cre; Ins2‐Dre^ER^; Rosa‐LSL‐RSR‐EGFP* mouse model, illustrating the dual‐recombinase system used to trace *Gcg^+^Ins^+^* bihormonal cells. B) Experimental timeline showing tamoxifen (TM) administration and subsequent embryonic tissue collection for lineage tracing of *Gcg^+^Ins^+^* cells. C) Immunofluorescence staining of EGFP, GCG, and INS in frozen pancreatic sections from *Gcg‐Cre; Ins2‐Dre^ER^; Rosa‐LSL‐RSR‐EGFP* mice at different embryonic stages. White arrowheads indicate EGFP^+^GCG^+^INS^+^ cells, and orange arrowheads mark EGFP^+^GCG^+^INS^−^ cells. Scale bars, 10 µm. D) PCA plot showing transcriptomic profiles of E14.5 β‐cells, α‐cells, and E14.5 and E16.5 EGFP^+^ cells traced at different points of pancreas development. Each dot represents a single cell; cell counts are shown in brackets. E) Violin plots showing *Ins1* and *Gcg* expression levels in the cells from (D). Black lines represent median expression levels. Each dot corresponds to a single cell. Statistical significance was assessed using the unpaired Wilcoxon rank‐sum test. F) Quantification of INS and GCG protein fluorescence intensities in cells from (C) at different embryonic stages. Cell counts are indicated in brackets. Data are presented as mean ± SEM (procedure‐defined units, p.d.u.). Statistical significance was determined using the unpaired Wilcoxon rank‐sum test. G) UMAP plot of scRNA‐seq data from the human fetal pancreas,^[^ ^1a^ ^]^ showing that *GCG*⁺*INS*⁺ cells cluster more closely with α‐cells than with β‐cells. H) Expression levels of *GCG* and *INS* in α‐, β‐, and *GCG*⁺*INS*⁺ cells from the human fetal dataset.^[^ ^1a^ ^]^ I,J) tSNE plot displaying cell types derived from hESCs (I), and tSNE‐based expression of marker genes in each cluster (J). EC: enterochromaffin‐like cell. Each dot represents a single‐cell. Cell counts are indicated in brackets. K) Violin plots of *INS* and *GCG* expression in cells shown in (I). Black lines denote median values. Each dot represents a single cell. Statistical comparisons were performed using the unpaired Wilcoxon rank‐sum test. L) Chromatin accessibility profiles of lineage‐specific transcription factors (*Arx* in α‐cells; *Mnx1* in β‐cells) and hormone genes (*Gcg*, *Ins1*, and *Ins2*) in α‐ and β‐cells at E16.5 and adult (P84). Left: cells are ranked by signal intensity (SI) across the shaded genomic region of each gene; cells with zero SI were excluded for *Arx*, *Mnx1*, and *Gcg*, but retained for *Ins1* and *Ins2*. Right: bar plots show mean SI within the shaded regions.

We then generated *Gcg‐Cre; Ins2‐Dre^ER^; Rosa‐LSL‐RSR‐EGFP* triple‐transgenic mice to trace the fate of *Gcg^+^Ins^+^* cells during embryonic development (**Figure** **4**A). TM administration at E13.5 or E14.0, followed by analysis of embryos at E14.5 or at E13.5 with follow‐up at E16.5 (Figure 4B). This approach allowed us to track labeled cells for 0.5, 1, or 3 days. Among embryos labeled for one day (TM E13.5→E14.5), immunostaining for EGFP, INS, and GCG confirmed successful labeling of *Gcg^+^Ins^+^* bihormonal cells in the triple‐transgenic line (Figure 4C).

Using FACS, we isolated EGFP⁺ bihormonal cells from E14.5 embryos, along with Ins1‐RFP⁺ and Gcg‐GFP⁺ monohormonal controls, for Smart‐seq3 scRNA‐seq (Figures S2B,S3C, Table S1, Supporting Information). Consistent with previous observations, GCN analysis revealed that EGFP^+^ cells from the triple‐transgenic strain were transcriptionally indistinguishable from α‐cells (Figure 4D; Figure S3D, Supporting Information). By examining *Ins1* and *Gcg* expression levels and fluorescent signals, we discovered that *Gcg* expression in bihormonal cells matched that in α‐cells, whereas *Ins1* expression was significantly lower than in β‐cells (Figure 4E). Immunofluorescence staining of E16.5 and E17.5 pancreas (after TM administration at E13.5 or E14.5) showed that EGFP signals predominantly colocalized with GCG, but not INS, indicating terminal differentiation into monohormonal α‐cells (Figure 4C). Fluorescence intensity analysis further confirmed that GCG levels in bihormonal cells were comparable to those in α‐cells, while INS levels remained lower than in β‐cells (Figure 4F).

These findings are consistent with a report by Villalba et al.,^[^ ^7c^ ^]^ which described the transient emergence of GCG^+^INS^+^ cells during early human pancreatic development (Carnegie stage 16–18), followed by their disappearance at later stages. However, due to limited molecular profiling, the exact identity of those cells—whether α‐like, β‐like, or progenitor‐like—remained unresolved.

To strengthen our cross‐species comparison, we reanalyzed scRNA‐seq data from the human fetal pancreas^[^ ^1a^ ^]^ and found that *GCG* ^+^ *INS* ^+^ cells also exhibited transcriptomic profiles closely aligned with α‐cells, mirroring our observations in the mouse model (Figure 4G,H).

We further examined a publicly available scRNA‐seq dataset derived from human embryonic stem cell (hESC)‐induced endocrine cells.^[^ ^21^ ^]^ At the final inductive stage, β‐cells constituted 11.2% of the total population, whereas *GCG* ^+^ *INS* ^+^ cells represented 23.8% (Figure 4I,J). *t‐*distributed stochastic neighbor embedding (tSNE) analysis revealed that most *GCG* ^+^ *INS* ^+^ cells clustered with α‐cells and exhibited lower levels of *INS* (Figure 4I–K), suggesting they are transcriptionally more similar to induced α‐cells. While these findings are consistent with our in vivo data, it is important to note that hESC‐derived endocrine cells may not fully recapitulate the metabolic, developmental, and signaling context of native human islets. Further comparisons across alternative differentiation protocols are needed to determine whether the α‐like identity of GCG⁺INS⁺ cells is universally conserved or context‐dependent.

In conclusion, through dual‐genetic tracing, scRNA‐seq, and cross‐species analyses, we demonstrate that *Gcg⁺Ins⁺* bihormonal cells predominantly exhibit α‐cell‐like transcriptional features and ultimately differentiate into Gcg⁺ monohormonal α‐cells during development.

### 2.6 Chromatin basis for insulin expression in α‐cells

We hypothesize that the presence of insulin expression in *Gcg⁺Ins⁺* bihormonal cells may result from transcriptional “leakage” in α‐cells during the competitive differentiation between α‐ and β‐cell lineages. To explore this possibility, we performed single‐nucleus ATAC‐seq (snATAC‐seq) on mouse embryonic endocrine cells using the *Ngn3‐Cre^ER^; Rosa‐RFP* lineage‐tracing model. Following tamoxifen injection at E15.5, RFP^+^ endocrine cells were isolated at E16.5 to capture newly differentiated populations. To place these findings in a broader developmental context, we also integrated previously published snATAC‐seq datasets from adult α‐ and β‐cells.^[^ ^22^ ^]^

Our analysis revealed that ≈0.53% of E16.5 α‐cells exhibited high chromatin accessibility at the *Ins1* locus, with signal intensity reaching ≈45% of that observed in β‐cells (Figure 4L). In these rare α‐cells, the chromatin profile at the insulin genes mirrored that of β‐cells, indicating that this accessibility pattern is specific rather than stochastic. Moreover, these α‐cells with elevated insulin gene accessibility were present at E16.5, with very few detected in adults, suggesting a developmentally restricted phenomenon (Figure 4L).

To validate the specificity of these findings, we examined chromatin accessibility at additional marker genes, including *Arx*, *Mnx1*, and *Gcg*. These control genes showed high chromatin accessibility in cell types where they are actively expressed (*Arx* and *Gcg* in α‐cells, and *Mnx1* in β‐cells) and significantly lower accessibility in low‐expressing populations (Figure 4L). This consistency across multiple loci supports the robustness and specificity of our snATAC‐seq data.

In summary, these results provide mechanistic insight into the transient insulin expression observed in developing α‐cells and suggest that chromatin‐level permissiveness at the insulin loci may underlie the emergence of *Gcg⁺Ins⁺* bihormonal cells during pancreatic development.

### 2.7 Assessment of Bihormonal Cells in Diabetic Mouse Models

Our dual‐reporter system enabled us to investigate whether bihormonal cells contribute to β‐cell regeneration under diabetic conditions. STZ, which selectively ablates β‐cells, is a widely used model in diabetes research. Previous studies have suggested that Ins⁺Sst⁺ bihormonal cells may serve as transient intermediates during transdifferentiation from δ‐cells to β‐cells in STZ‐treated mice.^[^ ^23^ ^]^ We hypothesized that such conversion events would involve the co‐expression of insulin and the originating endocrine hormone. To test this, we examined the presence of *Ins^+^Sst^+^* and *Gcg^+^Ins^+^* cells in *Ins1‐RFP; Sst‐BFP* and *Gcg‐GFP; Ins1‐RFP* mice following STZ administration, using IFC.

Two STZ treatment protocols were tested: a single high dose (200 mg kg^−1 ^) and multiple low doses (50 mg kg^−1 ^ day^−1^ for five consecutive days).^[^ ^24^ ^]^ In both adult (2‐month‐old) and juvenile (2‐week‐old) mice, the high‐dose (STZ‐high) regimen rapidly induced hyperglycemia within two days, with blood glucose levels exceeding the maximum detection threshold (34 mmol L^−1^) by days (D) 15 and 30 (Figure S4A, Supporting Information). Histological analysis revealed severely disrupted islet architecture and substantial β‐cell depletion by day 15 post‐treatment. The extent of residual β‐cells varied among islets, with some exhibiting near‐complete loss (Figure S4B,C, Supporting Information). Quantitative analysis revealed no significant differences in β‐cell numbers across D2, D15, and D30 post‐STZ treatment (Figure S4D, Supporting Information). The low‐dose protocol (STZ‐low) also induced hyperglycemia in adult mice. Although blood glucose levels remained below 34 mmol L^−1^ on D15, they exceeded this threshold by D30 (Figure S4E, Supporting Information), confirming successful diabetes induction.

**Figure 5:**
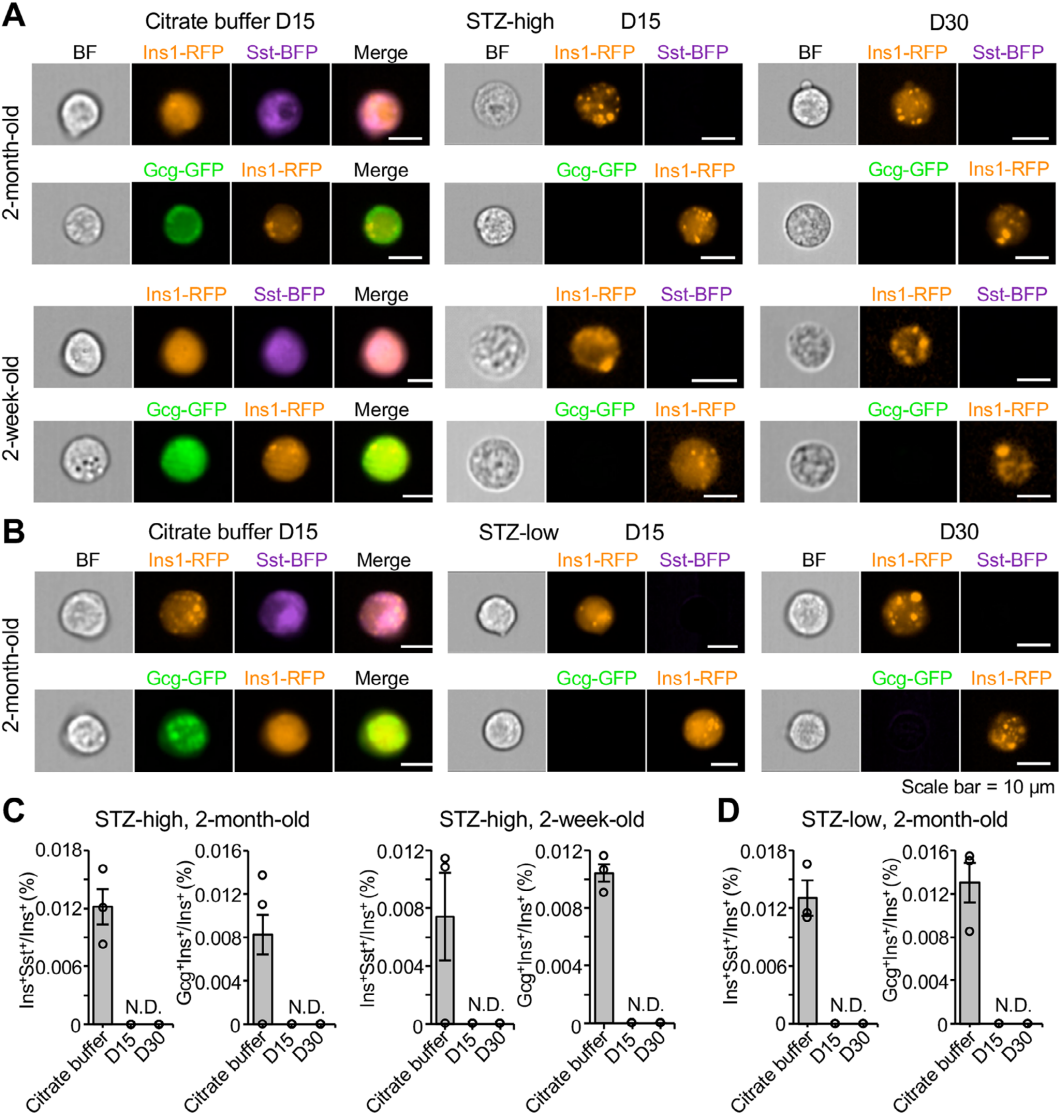
IFC analyses of bihormonal cells in STZ‐treated mice. A) Representative IFC images of bihormonal cells from the pancreata of *Ins1‐RFP; Sst‐BFP* and *Gcg‐GFP; Ins1‐RFP* mice treated with citrate buffer or high‐dose streptozotocin (STZ‐high) on day 15 (D15) and day 30 (D30). Scale bars, 10 µm. B) Representative IFC images of bihormonal cells from *Ins1‐RFP; Sst‐BFP* and *Gcg‐GFP; Ins1‐RFP* mice treated with citrate buffer or multiple low‐dose STZ (STZ‐low) on D15 and D30. Scale bars, 10 µm. C) Quantification of Ins1‐RFP⁺Sst‐BFP⁺ and Gcg‐GFP⁺Ins1‐RFP⁺ cells as a percentage of total Ins1‐RFP⁺ cells in mice treated or not treated with high‐dose STZ at different time points. N.D., not detected. Data are presented as mean ± SEM; *n* indicates the number of mice per group: citrate buffer, *n *= 3; STZ‐treated, *n *= 5. D) Quantification of Ins1‐RFP⁺Sst‐BFP⁺ and Gcg‐GFP⁺Ins1‐RFP⁺ cells as a percentage of total Ins1‐RFP⁺ cells in 2‐month‐old mice treated or not treated with low‐dose STZ at different time points. N.D., not detected. Data are presented as mean ± SEM; *n* indicates the number of mice per group: citrate buffer, *n *= 3; STZ‐treated, *n *= 5.

Flow cytometry analysis demonstrated significant β‐cell loss in both models, with the STZ‐low group retaining approximately four times more Ins1‐RFP⁺ cells than the STZ‐high group by day 15 and 30 (Figure S4F,G, Supporting Information). IFC analyses in both adult and juvenile mice confirmed that the majority of remaining RFP^+^ cells were monohormonal (**Figure** **5**A,B). Most dual‐fluorescent positive events were identified as cellular doublets (Figure S4H,I, Supporting Information), and genuine bihormonal cells were exceedingly rare in both STZ‐high and STZ‐low models (Figure 5C,D). Among over 500 islets examined microscopically, only two candidate INS⁺SST⁺ cells were identified in STZ‐treated samples (Figure S4J, Supporting Information). At four months post‐treatment, both Ins1‐RFP⁺Sst‐BFP⁺ and Gcg‐GFP⁺Ins1‐RFP⁺ cells remained virtually undetectable in STZ‐high‐treated mice (Figure S4K, Supporting Information).

To further assess bihormonal cells under extreme β‐cell depletion, we employed the *Pdx1‐Cre^ER^; Rosa‐DTA* model, which enables near‐complete β‐cell ablation when TM is administered at P15. Immunofluorescence analysis on D15 post‐TM injection revealed extensive β‐cell loss, comparable to or more severe than that observed in the STZ‐high model (Figure S5A, Supporting Information). To address potential concerns regarding *Pdx1* expression in δ‐cells, we performed single‐cell RT‐qPCR (scRT‐qPCR), confirming that *Pdx1* expression was rare in P15 α‐ and δ‐cells (Figure S5B, Supporting Information). Furthermore, lineage tracing using *Pdx1‐Cre^ER^*; *Rosa‐RFP* mice treated with TM at P15 showed RFP expression exclusively in INS^+^ β‐cells, not in SST^+^ cells (Figure S5C, Supporting Information), validating the specificity of the DTA system. Post‐ablation analysis demonstrated that SST⁺ δ‐cells were preserved, while INS⁺ β‐cells were depleted (Figure S5D, Supporting Information). Additional investigations revealed that INS^+^SST^+^ and GCG^+^INS^+^ cells were rarely detected at D2, D15, D30, and even four months after TM treatment (Figure S5E, Supporting Information).

Collectively, these results demonstrate that bihormonal cells are virtually undetectable in both STZ‐induced and DTA‐induced diabetic mouse models, suggesting they do not play a significant role in β‐cell regeneration in these contexts.

### 2.8 Conserved Endocrine Lineage Classification in Mouse and Human Embryos

Building on insights gained from the analysis of bihormonal cells, we extended our investigation to examine the broader developmental hierarchy of endocrine lineages in mouse and human embryonic pancreata. To this end, we performed GCN analysis to construct a refined, cross‐species classification of endocrine cell types.

Droplet‐based scRNA‐seq methodologies, notably those developed by 10× Genomics, allow for the simultaneous analysis of thousands of cells, offering comprehensive insight into cell identities and population heterogeneity. However, a key limitation of these platforms is doublet contamination, which arises when two or more cells are captured in a single droplet, leading to artificial transcriptomic profiles. Although computational strategies exist to reduce this artifact, it remains challenging to eliminate doublets entirely. To address these factors and ensure the robustness of our transcriptomic analysis, we excluded cells expressing more than one hormone, as these were more likely to represent doublets or bihormonal intermediates.

**Figure 6:**
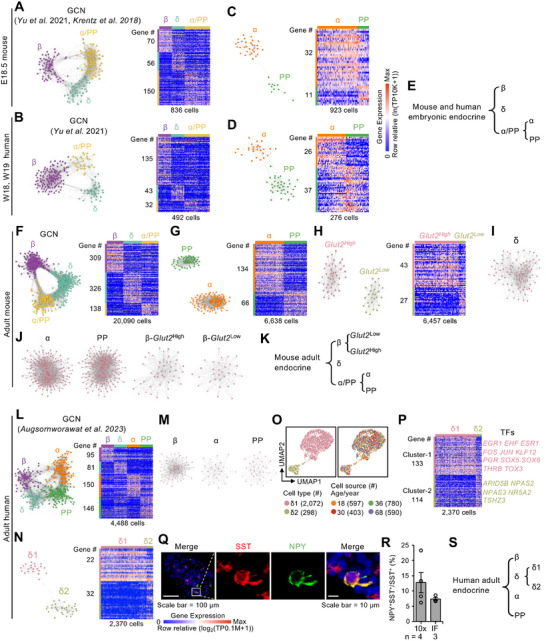
Comparative hierarchical classification of endocrine cells in mouse and human pancreata. A–D) GCN and heatmap analyses of embryonic endocrine cells: E18.5 mouse endocrine cells^[^ ^1^, ^25^ ^]^ (A) and W18 and W19 human endocrine cells^[^ ^1a^ ^]^ (B) reveal three major lineages—β‐, δ‐, and α/PP‐cells. Further resolution of α/PP cells in mouse (C) and human (D) embryos shows transcriptional separation of α‐ and PP‐cells. E) Schematic summarizing the hierarchical organization of endocrine cell types in embryonic mouse and human pancreata. F–H) GCN and heatmap analyses of adult (P60) mouse islets showing gene modules for β‐, δ‐, and α/PP‐cells (F), further separation of α‐ and PP‐cells (G), and β‐cell subtypes *Glut2* ^High^ and *Glut2* ^Low^ (H). Gene counts per module are indicated. Each column represents a single cell; each row represents a gene. I,J) GCN analyses of δ‐cells (I) and α‐, PP‐, *Glut2* ^High^, and *Glut2* ^Low^ β‐cells (J) in P60 mouse islets. K) Schematic summary of the hierarchical classification of adult mouse islet endocrine cells. L–N) Analyses of adult human pancreatic islets based on data from Augsornworawat et al.^[^ ^27^ ^]^ GCN and heatmap showing four endocrine clusters—α‐, PP‐, β‐, and δ‐cells (L); GCNs for β‐, α‐, and PP‐cells (M); and GCN/heatmap comparison of δ1‐ and δ2‐cells (N). O) UMAP plots showing δ‐cell subtypes and their sample origins. Cell counts are indicated in brackets. P) Heatmap of DEGs between δ1‐ and δ2‐cells, including gene cluster sizes and key transcription factors (TFs). Q) Immunofluorescence staining of SST and NPY in adult human islets. Scale bars, 100 µm (left), 10 µm (right). R) Quantification of NPY^+^SST^+^ δ2‐cells as a percentage of total SST^+^ δ‐cells based on scRNA‐seq (10× Genomics) and immunofluorescence (IF) data. *n* indicates the number of biological replicates. S) Schematic summary of the hierarchical classification of endocrine cells in adult human islets.

We conducted a comparative analysis of endocrine cells during embryonic development in humans and mice using 10× Genomics v2 scRNA‐seq data.^[^ ^1^, ^25^ ^]^ GCN analysis was performed on mouse samples at E18.5 and human samples at post‐conception weeks 18 (W18) and 19 (W19). In both species, three major GCN‐defined endocrine clusters were identified, corresponding to β‐, δ‐, and a combined α/PP‐cell population (**Figure** **6**A,B; Figure S6A–D, Table S3, Supporting Information). Further resolution of the α/PP group revealed distinct gene modules specific to α‐ and PP‐cells in each species (Figure 6C,D; Table S3, Supporting Information).

These results suggest that the fundamental structure of endocrine lineage specification is broadly conserved between mice and humans during embryogenesis (Figure 6E).

### 2.9 Distinct Hierarchical Classification of Endocrine Lineages in Adult Mice and Humans

To explore the organizational structure of adult endocrine cell types, we performed scRNA‐seq and GCN analysis on pancreatic islet cells from both mice and humans. In mice, fluorescently labeled endocrine cells from P60 islets were isolated via FACS and profiled using the 10× Genomics v3 platform (Figure S6E, Supporting Information). Following quality control and the exclusion of *Spi1* ^+^ immune cells and *Kdr* ^+^ mesenchymal cells, a total of 22543 *Neurod1* ^+^ endocrine cells were retained for downstream analysis (Figure S6F,G, Table S4, Supporting Information). Uniform manifold approximation and projection (UMAP) analysis revealed 10 distinct clusters, including four monohormonal endocrine types (β‐, α‐, δ‐, and PP‐cells) and six dual‐hormone populations (Figure S6H,I, Supporting Information). Given the rarity of bona fide bihormonal cells in adult islets, the presence of dual‐marker‐positive cells is likely influenced by biological factors such as physical interactions and shared developmental origins. For instance, δ‐cells possess filamentous projections that may increase the likelihood of forming doublets during FACS sorting. Similarly, α‐ and PP‐cells originate from a common progenitor, and residual plasticity between the two lineages may account for occasional marker co‐expression. To ensure robust transcriptomic analysis, we excluded all dual‐hormone‐expressing cells, resulting in a refined dataset of 20 090 monohormonal cells.

GCN analysis of this monohormonal dataset identified three major transcriptional clusters corresponding to β‐, δ‐, and a combined α/PP‐cell lineage (Figure 6F, Table S3, Supporting Information). Further resolution of the α/PP group revealed distinct gene modules associated with α‐cells and PP‐cells, respectively (Figure 6G, Table S3, Supporting Information). Additional GCN analysis uncovered two β‐cell subpopulations—*Glut2* ^High^ and *Glut2* ^Low^—which are consistent with previously reported metabolic heterogeneity among β‐cells^[^ ^14^, ^26^ ^]^ (Figure 6H, Table S3, Supporting Information). Notably, each of the δ‐, α‐, PP‐, *Glut2* ^High^‐, and *Glut2* ^Low^‐cell populations exhibited distinct GCNs (Figure 6I,J), indicating intrinsic coexpression stability. This stratified GCN analysis provides a comprehensive hierarchical classification of mouse endocrine cells (Figure 6K).

This approach was extended to adult human endocrine cells using single‐nucleus RNA‐seq (snRNA‐seq) data generated with the 10× Genomics platform, as reported by Augsornworawat et al.^[^ ^27^ ^]^ After excluding bihormonal cells, the dataset comprised 23985 endocrine cells, including 10858 β‐cells, 2370 δ‐cells, 9635 α‐cells, and 1122 PP‐cells, with an average of ≈3500 genes detected per cell (Figure S7A–C, Supporting Information). To address sample size biases, all cell types were downsampled to match the smallest group—PP‐cells (1122 cells). GCN analysis revealed four clearly defined transcriptional clusters corresponding to β‐, δ‐, α‐, and PP‐cells (Figure 6L, Table S3, Supporting Information). Notably, GCNs for β‐, α‐, and PP‐cells showed no further internal substructure (Figure 6M). However, the δ‐cell population split into two distinct clusters, δ1 and δ2 (Figure 6N,O; Figure S7D, Tables S3,S5, Supporting Information). Differential expression analysis identified 247 DEGs between δ1 and δ2, including neuropeptide Y (*NPY*), which was specifically enriched in δ2‐cells (Figure 6P; Figure S7D, Table S5, Supporting Information). Gene ontology (GO) enrichment analysis revealed that δ1‐cells were associated with hormone secretion pathways, while δ2‐cells were enriched in genes related to cell–cell interactions (Figure S7E, Table S5, Supporting Information). Immunofluorescence staining confirmed the presence of NPY in a small subset of SST^+^ δ‐cells, establishing a δ1:δ2 ratio of ≈9:1 in adult human islets (Figure 6Q,R). Notably, *Npy* expression was not detected in adult mouse δ‐cells (Figure S7F, Supporting Information).

To gain insight into the developmental origins of these subtypes, we reanalyzed scRNA‐seq data from a 13‐month‐old infant pancreas.^[^ ^21^ ^]^ Although *NPY* expression was absent at this age, transcriptomic clustering and differential expression of other marker genes enabled the classification of infant δ‐cells into δ1‐ and δ2‐like populations (Figure S7G,H, Supporting Information), suggesting that δ‐cell heterogeneity emerges during late gestation and is evident by early postnatal stages. However, due to ethical limitations, fetal human samples from this window remain inaccessible.

To further investigate the epigenetic basis of δ‐cell heterogeneity, we analyzed snATAC‐seq data from Augsornworawat et al.^[^ ^27^ ^]^ Both independent snATAC‐seq analysis and integrated snRNA‐seq/snATAC‐seq analysis consistently identified δ1 and δ2 populations (Figure S7I, Supporting Information). Chromatin accessibility profiling revealed higher promoter accessibility of cluster‐1 genes in δ1‐cells, while δ2‐cells exhibited greater accessibility at the *NPY* locus and other cluster‐2 genes (Figure 6P; Figure S7J,K, Supporting Information). Differential accessible peak (DAP) analysis revealed 968 differentially accessible regions, and motif enrichment analysis revealed that RFX family motifs were enriched in δ1‐cells, whereas ARID3A, EGR1, and KLF15 motifs were more accessible in δ2‐cells (Figure S7L,M, Supporting Information). Overall, our findings reveal transcriptional and epigenetic heterogeneity within δ‐cells, highlighting the existence of two molecularly distinct subtypes in the human islet.

To further validate our findings, we analyzed an additional adult human scRNA‐seq dataset from Tritschler et al.,^[^ ^28^ ^]^ generated using the 10× Genomics v2 platform. After quality control and cell annotation, the dataset included 3681 β‐cells, 1833 δ‐cells, 4047 α‐cells, and 101 PP‐cells (Figure S8A,B, Supporting Information), with an average number of ≈3000 genes detected per cell (Figure S8C, Supporting Information). After downsampling to 101 cells per group, GCN analysis revealed four well‐separated endocrine lineages, in contrast to the partial overlap of α‐ and PP‐cells observed during embryonic development (Figure 6B; Figure S8D, Supporting Information).

We also analyzed a third adult human islet dataset from van Gurp et al.,^[^ ^11b^ ^]^ generated using the 10× Genomics v3 platform. After excluding bihormonal cells, we performed GCN analysis on the remaining 10711 endocrine cells (Figure S8E,F, Supporting Information). Despite lower gene detection depth compared to Augsornworawat et al.^[^ ^27^ ^]^ (Figure S8G, Supporting Information), both the four‐lineage architecture and the δ1/δ2 substructure were recapitulated (Figure S8H–J, Tables S3,S5, Supporting Information), confirming the robustness of our classification.

For cross‐species comparison, we downsampled 1122 cells from each mouse endocrine cell type and performed GCN analysis under the same conditions (Figure S8K, Supporting Information). This strategy ensured consistency in our comparative analysis of human and mouse datasets.

In summary, our comprehensive analysis demonstrates that while mouse and human islets share four canonical endocrine lineages (β‐, δ‐, α‐, and PP‐cells), their internal transcriptional architecture diverges. Human islets show clearer separation between α‐ and PP‐cells and a human‐specific subdivision of δ‐cells into δ1 and δ2, whereas mouse islets display persistent α/PP overlap and β‐cell heterogeneity. These findings underscore fundamental species‐specific differences in adult endocrine organization (Figure 6K,S).

### 2.10 Cell Type‐Specific Transcriptome Differences in Adult Mouse and Human Endocrine Cells

To investigate interspecies differences in endocrine cell identity, we performed a refined comparative analysis of DEGs in adult mouse and human islets using a relatively high‐quality snRNA‐seq dataset.^[^ ^27^ ^]^ To reduce potential artifacts from cell contamination, we first excluded all polyhormonal cells. Focusing initially on DEGs between α‐ and β‐cells, we observed that the number of DEGs plateaued when more than 1000 cells were sampled (Figure S9A, Supporting Information). Based on this, we normalized cell numbers across endocrine types by downsampling each group to match the least abundant population—human PP‐cells (1122 cells)—to ensure consistency across datasets.

**Figure 7:**
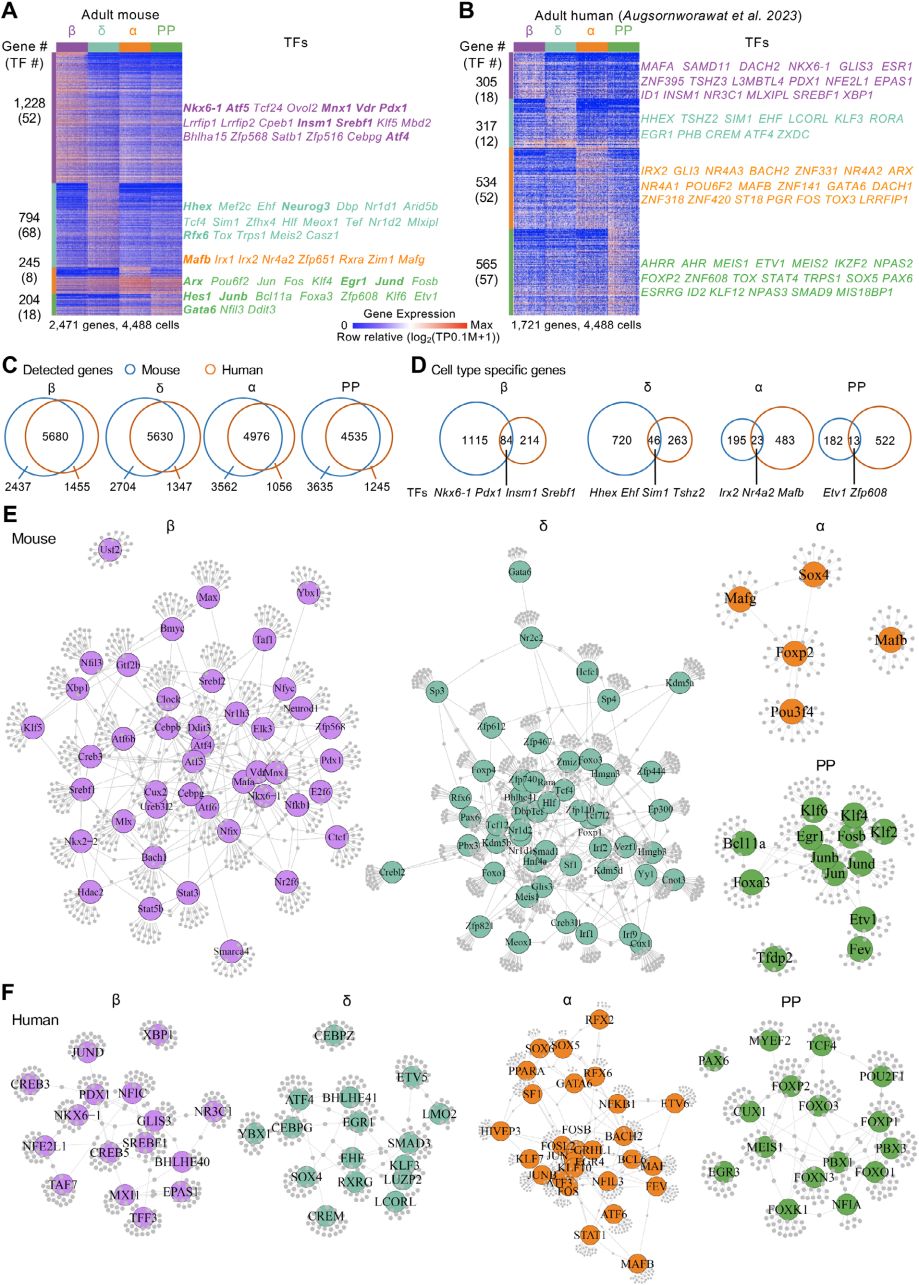
Comparative analysis of DEGs and gene regulatory networks in adult mouse and human endocrine cells. A,B) Heatmaps showing DEGs in adult mouse (A) and human (B) pancreatic endocrine cells. Each column represents a single cell, and each row a gene. For each cluster, the number of DEGs and up to 20 transcription factors (TFs), ranked by fold change, are annotated. TFs previously studied in pancreatic endocrine cells are highlighted in bold in the mouse dataset. DEGs were identified using the unpaired Wilcoxon rank‐sum test with the following parameters: adjusted *P *≤ 10⁻⁵, log₂(fold change) ≥ 0.5, pct.1 ≥ 0.35. TF information was obtained from AnimalTFDB v3.0.^[^ ^45^ ^]^ C) Venn diagrams showing the overlap of homologous genes expressed in each endocrine cell type between mouse and human. Genes were included if expressed in at least 15% of cells in each type. Gene counts are indicated. D) Venn diagram showing the overlap of homologous TFs expressed in mouse and human endocrine cell types. E,F) Gene regulatory networks (GRNs) for β‐, δ‐, α‐ and PP‐cells in mouse (E) and human (F). Colored nodes represent transcriptional regulators (TFs), while gray nodes denote their predicted target genes. Edges indicate regulatory interactions inferred from network analysis.

This DEG analysis identified distinct molecular signatures for each endocrine cell type. In total, we identified 2471 DEGs in mice and 1721 in humans, including numerous TFs (**Figure** **7**A,B, Table S6, Supporting Information). In mice, β‐cells showed the most unique genes (1228), whereas in humans, α‐ and PP‐cells displayed more DEGs than β‐cells (Figure 7B), suggesting species‐specific variation in transcriptional specialization.

Although there was a notable overlap of homologous genes across endocrine cell types between species (Figure 7C), cell type–specific gene expression profiles were largely non‐overlapping (Figure 7D, Table S6, Supporting Information). In addition, we observed species‐specific differences in TF centrality within GCNs, suggesting divergence in regulatory hierarchy (Figure S9B,C, Supporting Information). For example, *Pdx1* and *Nkx6‐1* showed higher network centrality in mouse β‐cells than in their human counterparts, while *Arx* was more central in mouse PP‐cells but shifted toward α‐cells in human islets (Figure S9B,C, Supporting Information).

To further explore regulatory architecture, we performed single‐cell regulatory network inference and clustering (SCENIC)‐based regulon analysis, which identified 44, 50, 5, and 13 cell type–specific regulons in mouse β‐, δ‐, α‐, and PP‐cells, respectively, and 16, 28, 16, and 16 regulons in the corresponding human cell types (Figure 7E,F; Figure S9D,E, Supporting Information). Although some TFs such as *NKX6‐1*, *PDX1*, and *MAFB* were conserved across species, their regulon compositions and target networks diverged substantially, highlighting species‐specific wiring of gene regulatory circuits (Figure 7E,F).

Taken together, these findings reveal substantial interspecies differences in both transcriptional output and regulatory network architecture within endocrine cells. They underscore fundamental distinctions between the pancreatic endocrine systems of mice and humans, which may influence how endocrine cell types differentiate and function across species.

## 3 Discussion

Previous studies employing immunoimaging and scRNA‐seq have reported the existence of bihormonal cells in both mouse^[^ ^1^, ^3^, ^8^, ^9^, ^11^ ^]^ and human pancreata.^[^ ^7^, ^10^, ^11^ ^]^ However, the exact prevalence, cellular identities, and functional relevance of these bihormonal cells remain unclear, largely due to technical limitations such as fluorescence signal spillover and challenges in accurate cell sorting.

In the present study, we used a combination of genetically labeled reporter mouse strains, IFC, and single‐cell transcriptomics to systematically characterize bihormonal cells across multiple developmental stages. Our analysis revealed that the majority of dual‐fluorescent positive cells isolated by FACS were in fact cellular doublets, rather than genuine bihormonal cells.

We identified six types of bihormonal cell combinations within pancreatic islets, all of which were rare, except for a relatively enriched population of *Gcg‐GFP^+^Ppy‐mNeptune^+^* cells. The differential doublet formation rates across endocrine cell types likely stem from their distinct physical interactions and structural properties. For instance, β‐cells, being the majority, had a higher probability of forming doublets with neighboring cells. δ‐cells, which possess elongated filamentous processes, frequently made contact with adjacent cells, increasing the likelihood of doublet formation. In contrast, α‐ and PP‐cells exhibited fewer interactions, possibly due to their spatial segregation between dorsal and ventral pancreatic regions.^[^ ^1a^ ^]^

Our scRNA‐seq analysis revealed that *Gcg‐GFP^+^Ppy‐mNeptune^+^* bihormonal cells closely resembled either α‐ or PP‐cells and lacked unique gene expression profiles. A recent study by Perez‐Frances et al. identified *Gcg^+^Ins^+^* bihormonal cells in the fetal mouse pancreas, suggesting that embryonic GCG‐expressing cells may give rise to adult INS⁺ cells.^[^ ^3c^ ^]^ However, conventional lineage‐tracing tools may not specifically label true bihormonal cells, as endocrine progenitors can transiently express multiple hormones during their gradual differentiation into INS⁺ β‐cells.^[^ ^1a^ ^]^ Using our dual‐genetic‐tracing system in combination with scRNA‐seq, we confirmed that *Gcg⁺Ins⁺* cells are transcriptionally indistinguishable from α‐cells, indicating that these cells are not a distinct lineage but likely represent a transient state within the α‐cell developmental trajectory. We propose that insulin expression in these cells reflects a temporary transcriptional overlap during the bifurcation of α‐ and β‐cell fates, possibly due to asynchronous chromatin closure and activation. This interpretation is supported by our snATAC‐seq analysis of embryonic and adult α‐ and β‐cells, which revealed residual chromatin accessibility at insulin loci in α‐cells. Therefore, Gcg⁺Ins⁺ cells may arise as byproducts of rapid lineage transitions rather than intermediates with stable regulatory identity. Supporting this, multihormonal cells—particularly GCG⁺INS⁺ cells—frequently appear in in vitro islet induction protocols and subsequently resolve into monohormonal α‐cells after transplantation.^[^ ^29^ ^]^

Perturbations in gene regulation during the competitive differentiation of endocrine cells can result in the simultaneous expression of multiple hormones, which may not be governed by the specific signaling pathways traditionally associated with endocrine lineage commitment. As differentiation progresses, bihormonal cells typically resolve into monohormonal types by silencing one hormone program. Notably, certain bihormonal populations, such as *Gcg^+^Ppy^+^* cells in mice, may persist into adulthood. However, their low prevalence and lack of distinct transcriptomic identity suggest that such cells contribute minimally to overall islet function.

Our study identified Gcg^+^Ppy^+^ cells as the most prevalent bihormonal population in adult mouse islets. Transcriptomic and GCN analyses revealed that these cells closely resemble either α‐cells or PP‐cells, without forming a distinct endocrine subtype. This finding is consistent with previous reports, including that by Perez‐Frances et al.,^[^ ^9a^ ^]^ which showed that *Ppy*‐expressing cells frequently coexpress other islet hormones, and that Gcg^+^Ppy^+^ cells are the most abundant among Ppy^+^ bihormonal populations. Moreover, their lineage‐tracing data suggest that some adult Gcg^+^Ppy^+^ cells originate from embryonic counterparts, indicating that they may represent a stable state. Collectively, these observations indicate that the persistence of Gcg⁺Ppy⁺ cells likely reflects a shared developmental origin from a common progenitor downstream of endocrine precursors, rather than a distinct or plastic lineage.

As an application of our findings, we examined the behavior of bihormonal cells under both physiological and pathological conditions. In STZ‐induced and DTA‐induced diabetic mouse models, we found virtually no evidence of insulin‐coexpressing bihormonal cells, suggesting that such cells do not significantly contribute to β‐cell regeneration. Given their extremely low abundance and lack of expansion following β‐cell injury, it is unlikely that bihormonal cells play a substantial role in islet remodeling or disease progression.

Nevertheless, we acknowledge the complexity of β‐cell regeneration, which may involve dedifferentiation or transdifferentiation of other endocrine cells. Although we did not observe transitions from monohormonal to bihormonal states in our models, such events could occur at low frequencies or through intermediate states, such as hormone‐negative progenitor‐like cells, that may evade detection. Alternatively, β‐cell regeneration may primarily result from the proliferation of residual β‐cells, rather than through lineage conversion. It is also possible that regeneration involving a bihormonal intermediate is minimal or absent under the experimental conditions used.

We applied GCN analysis to scRNA‐seq data to refine the hierarchical classification of endocrine cells in both mouse and human islets. To avoid confounding effects from ambiguous gene expression, bihormonal cells were excluded from GCN analysis. In mouse islets, we identified three primary transcriptional groups: β‐cells, δ‐cells, and a combined α/PP‐cell population. In contrast, human islets showed four well‐separated endocrine: β‐, δ‐, α‐, and PP‐cells. Interestingly, α‐ and PP‐cells initially formed a shared group during human embryonic development but became distinct in adulthood, suggesting a more complex differentiation trajectory in humans than in mice. This developmental divergence may contribute to the broader diversity of cell type‐specific genes observed in human α‐ and PP‐cells. Furthermore, our systematic investigation of endocrine cell differentiation pathways in mice and humans revealed that, unlike the distinct developmental origins of β‐ and δ‐cells, α‐ and PP‐cells in both species emerge from common intermediate precursors.^[^ ^1a^ ^]^ This conclusion is supported by our GCN results, which showed extensive transcriptional overlap and shared regulatory modules between α‐ and PP‐cells. Moreover, the differentiation window for α‐ and PP‐cells is shorter in mice than in humans,^[^ ^1a^ ^]^ potentially contributing to the less distinct transcriptional separation between these cell types in mouse islets.

Although several studies have reported heterogeneity among human β‐cells—spanning glucose responsiveness, insulin secretion, maturation, proliferation, and gene expression profiles^.^ ^30^ ^]^ —these findings have often been inconsistent. Our GCN‐based approach, which is less affected by minor fluctuations in gene expression, revealed a surprisingly uniform regulatory network among human β‐cells, suggesting a more stable and homogeneous identity than previously assumed. In contrast, our analysis uncovered two transcriptionally and epigenetically distinct δ‐cell subpopulations in human islets, designated δ1 and δ2. This δ‐cell heterogeneity was not observed in mouse islets, highlighting an additional layer of complexity unique to the human endocrine system. Further research is warranted to elucidate the physiological roles and developmental origins of these δ‐cell subtypes.

In a recent study, Tritschler et al. compared adult human and mouse islet endocrine cells using the 10× Genomics v2 platform.^[^ ^28^ ^]^ Building on this, we conducted a more rigorous DEG analysis using balanced sample sizes across cell types from both species. Our results revealed limited overlap in cell type‐specific gene signatures between humans and mouse islets, indicating fundamental differences in the regulatory pathways that govern endocrine cell differentiation and maturation. This discrepancy underscores the challenges of translating mechanistic insights from mouse models to human biology, particularly in the context of diabetes and other endocrine disorders. It further highlights the importance of accounting for interspecies variation when applying findings from mouse studies to guide stem cell–derived islet differentiation protocols in human systems.

In summary, this study systematically characterized the identity, developmental dynamics, and lineage potential of bihormonal cells in mammalian pancreatic islets. By integrating dual‐reporter mouse models, IFC, single‐cell transcriptomics, and GCN analysis, we refined the classification of islet endocrine cell types in both species. Our findings provide valuable insight into the nature of bihormonal cells and reveal species‐specific features of islet architecture, offering important implications for both basic islet biology and translational diabetes research.

## 4 Experimental Section

### Mice

The transgenic mouse strains *Ins1‐RFP*,^[^ ^15^ ^]^ *Ins1‐BFP*, *Gcg‐GFP*,^[^ ^11g^ ^]^ *Sst‐BFP*,^[^ ^1a^ ^]^ and *Ppy‐mNeptune* ^[^ ^1a^ ^]^ were utilized for the enrichment of diverse pancreatic endocrine cells. To enrich embryonic endocrine cells for snATAC‐seq, the *Ngn3‐Cre^ER^* ^[^ ^31^ ^]^ strain was used. Additionally, the *Pdx1‐Cre^ER^*,^[^ ^31^ ^]^ *Gcg‐Cre*,^[^ ^32^ ^]^ *Ins2‐Dre^ER^* ^[^ ^14b^ ^]^ and *Rosa‐LSL‐RSR‐EGFP* strains were used for validation of this reporter mouse model and for lineage tracing of *Gcg^+^Ins^+^* bihormonal cells. All transgenic mouse strains were generated and maintained on the C57BL/6 genetic background.

All animals were housed in specific pathogen‐free animal facilities under a 12‐hour light/12‐hour dark cycle at Peking University. All experimental procedures were conducted in accordance with the guidelines approved by the Institutional Animal Care and Use Committee (IACUC) of Peking University Health Science Center (Approval No. BCAA0320).

The *Ins1‐BFP* and *Rosa‐LSL‐RSR‐EGFP* mouse strains were generated via CRISPR/Cas9‐mediated homologous recombination by GemPharmatech Co., Ltd. In vitro‐transcribed sgRNAs and donor constructs were co‐injected with Cas9 mRNA into fertilized C57BL/6JGpt embryos. The donor templates were cloned into a pMD18‐T backbone for homologous recombination. Founder (F0) mice were screened by PCR and validated by Sanger sequencing, and correctly targeted F0 animals were bred with C57BL/6JGpt mice to establish stable F1 lines. For the *Ins1‐BFP* strain, a *P2A‐BFP* cassette was inserted upstream of the translation stop codon (TAA) in exon 2 of the *Ins1* gene using the sgRNA: 5′‐CCGGGCCACCTCCAACGCCA‐3′. For the *Rosa‐LSL‐RSR‐EGFP* strain, the CAG promoter–loxP–stop–loxP–rox–stop–rox–EGFP cassette was inserted into the *Rosa26* locus using two sgRNAs: 5′‐GGCAGGCTTAAAGGCTAACC‐3′ and 5′‐AGTCTTCTGGGCAGGCTTAA‐3′. Genotypic was performed by PCR using the following primers: *Rosa‐LSL‐RSR‐EGFP* (Forward 5′‐CCCAAAGTCGCTCTGAGTTGTTA‐3′; Reverse 5′‐TCAATGGAAAGTCCCTATTGGCGT‐3′), and *Ins1‐BFP* (Forward 5′‐TTCTACACACCCAAGTCCCGC‐3′; Reverse 5′‐CTTAATCAGCTCGCTCATAGGAC‐3′).

The detection of a vaginal plug was marked as embryonic day 0.5 (E0.5). Tamoxifen was dissolved in sunflower seed oil at 20 mg mL^−1^ and administered intraperitoneally to the mice via a single dose of 0.1 mg g^−1^ body weight in accordance with the designated time points for each specific experimental protocol. Specifically: 1) *Pdx1‐Cre^ER^; Rosa‐LSL‐RSR‐EGFP*, *Ins2‐Dre^ER^; Rosa‐LSL‐RSR‐EGFP*, and *Pdx1‐Cre^ER^; Ins2‐Dre^ER^; Rosa‐LSL‐RSR‐EGFP* mice received a single tamoxifen dose prior to tissue collection. 2) For embryonic lineage tracing, tamoxifen was administered to *Gcg‐Cre; Ins2‐Dre^ER^; Rosa‐LSL‐RSR‐EGFP* pregnant females at: E13.5, with tissue collected at E14.5 and E16.5; E14.0, with tissue collected at E14.5; and E14.5, with tissue collected at E17.5. 3) *Ngn3‐Cre^ER^; Rosa‐RFP* pregnant females received tamoxifen at E15.5, and RFP⁺ endocrine cells were isolated at E16.5. 4) For postnatal studies, *Pdx1‐Cre^ER^; Rosa‐DTA* and *Pdx1‐Cre^ER^; Rosa‐RFP* mice were injected with tamoxifen at postnatal day 15 (P15), followed by immunofluorescence analysis 2, 15, and 30 days (D2, D15, D30) and 4 months later.

### Primary Human Islet Samples

Primary human islet tissue samples were obtained from the Department of Hepatobiliary Surgery of Peking University People's Hospital. Islets from three adult male patients were processed for immunofluorescence analysis. All procedures were conducted in strict accordance with protocols approved by the Peking University Institutional Review Board (PU‐IRB; certificate #IRB00001052‐22184). Informed consent was obtained from all patients prior to tissue collection.

### Single‐Cell Isolation and Flow Cytometry—Isolation of Adult Mouse Islets

Adult mice were humanely euthanized, and the abdominal cavity was exposed.^[^ ^16^, ^33^ ^]^ The duodenum was clamped at both ends near the entry of the common bile duct. A prechilled solution of 1  mg mL^−1^ collagenase P (Roche, 11213873001) was injected into the common bile duct to inflate the pancreas. The inflated pancreas was then incubated in a 37 °C water bath for 6–8 min with gentle shaking. Islets were manually picked under a microscope and subsequently digested into single‐cell suspensions using 0.25% trypsin for 5 min at 37 °C.

### Single‐Cell Isolation and Flow Cytometry—Digestion of the Embryonic and Perinatal Mouse Pancreas

Pancreata from E14.5 to P1 embryos were dissected and digested with 1 mg mL^−1^ collagenase P for 3–5 min, and the duration adjusted based on the organ size.^[^ ^11g^ ^]^ The tissue was then treated with 0.25% trypsin for 5 min at 37 °C to ensure complete dissociation into single cells.

### Single‐Cell Isolation and Flow Cytometry—Flow Cytometry Sorting

After enzymatic digestion, single‐cell suspensions were sorted using a BD FACSAria Fusion flow cytometer. Endogenous fluorescent protein reporters were used to identify and isolate specific endocrine cell populations.

### Immunofluorescence and Microscopy

Pancreata from P60 mice were fixed in 4% paraformaldehyde for 12 h at 4 °C. Embryonic pancreas samples were fixed for 6 h under the same conditions. After fixation, the tissues were washed three times with phosphate‐buffered saline (PBS). For cryosectioning, samples were sequentially dehydrated in 15% and 30% sucrose, embedded in OCT compound (Thermo Fisher, 6502), and rapidly frozen on dry ice. Frozen tissues were sectioned at a thickness of 5 µm using a cryostat.

Human islets were fixed in 4% paraformaldehyde for 6 h, washed three times with PBS, dehydrated in ethanol following standard protocols, embedded in paraffin, and sectioned into 5‐µm‐thick slices.

For immunofluorescence staining, tissue sections were incubated with the following primary antibodies: anti‐insulin (Abcam, ab7842, 1:500), anti‐glucagon (Millipore, AB932, 1:500), anti‐somatostatin (Santa Cruz, sc‐74556, 1:200), and anti‐neuropeptide Y (ImmunoStar, 22940, 1:200). Secondary antibodies included goat anti‐guinea pig Alexa Fluor 488 (Abcam, ab150185, 1:500), donkey anti‐rabbit Alexa Fluor 594 (Invitrogen, A21207, 1:500), and donkey anti‐mouse Alexa Fluor 488 (Invitrogen, A21202, 1:500). Confocal images were captured using a Leica TCS SP8 microscope. For pancreas sections with dual‐fluorescence labeling, direct imaging was conducted without additional staining.

For cell‐level imaging, FACS‐sorted cells were seeded onto poly‐*L‐*lysine–coated glass slides for 20 min at room temperature and then fixed with 4% paraformaldehyde for 15 min. Fluorescence images were acquired directly using a Leica TCS SP8 microscope.

### Imaging Flow Cytometry (IFC) Analysis

Samples from mice expressing various fluorescent reporters at different developmental stages were analyzed using IFC. Single‐cell suspensions were prepared according to the protocols described above. These suspensions were loaded into the Amnis ImageStreamX Mark II system, which captured high‐resolution images of individual cells while simultaneously recording their fluorescent signals. This approach enabled the identification of double‐positive cells—those expressing two distinct fluorescent markers—reflecting potential bihormonal identity.

Data were processed and analyzed using the ImageStream data exploration and analysis software, which integrates image features with quantitative fluorescence metrics. This approach enabled the identification and characterization of specific cell populations within the sample based on their fluorescence profiles.

### In Vivo Streptozotocin (STZ) Treatment

STZ was freshly prepared at a concentration of 10 mg mL^−1^ in 50 mm sodium citrate buffer and used within 15 min to ensure stability. Two distinct treatment protocols were employed:

1. High‐dose STZ protocol: Adult mice were fasted for 16 h and subsequently received a single intraperitoneal injection of STZ at 200 mg kg^−1 ^ body weight.
2. Multiple low‐dose STZ protocol: Mice were fasted for 6 h daily and administered 50 mg kg^−1 ^ STZ intraperitoneally for five consecutive days.

Control mice received an equivalent volume of sodium citrate buffer and underwent identical fasting and injection procedures to control for potential confounding effects of treatment conditions. This design allowed the authors to isolate the specific impact of STZ on pancreatic β‐cell integrity and function.

### Insulin Treatment

To prevent mortality during long‐term experiments, mice that developed hyperglycemia (blood glucose levels > 20 mmol L^−1^) were treated with subcutaneous insulin implants (LinBit).

### Statistical Analysis

All quantitative data were obtained from a minimum of three biological replicates per experimental condition. Statistical analyses were performed using GraphPad Prism software. Data were presented as mean ± standard error of the mean (SEM). Comparisons between two independent groups with unequal variances were conducted using Welch's *t*‐test. A *P*‐value of less than 0.05 (*P *< 0.05) was considered statistically significant. Detailed descriptions of the computational and statistical methods used for scRNA‐seq data analysis are provided in the corresponding sections of the manuscript.

### Single‐Cell RNA Sequencing (scRNA‐seq)

To meet the combined demands of transcriptomic precision and throughput, two complementary scRNA‐seq approaches were employed.

For high‐resolution analysis, the Smart‐seq3 protocol was used.^[^ ^18^ ^]^ Single cells were collected using two methods: 1) direct sorting into 384‐well plates preloaded with lysis buffer, and 2) manual extraction from FACS‐purified populations using a mouth pipette—particularly useful for targeting specific cell types with high accuracy. Libraries were generated from the captured cells and sequenced on an MGI DNBSEQ‐T7 platform using paired‐end 100‐bp reads (PE100), ensuring high coverage and read quality.

For high‐throughput profiling, droplet‐based scRNA‐seq was performed on P60 mouse islet endocrine cells using the Chromium Single Cell 3′ Reagent Kit v3 (10× Genomics). At least two biological replicates were included for each of the five dual‐reporter mouse lines analyzed. Libraries were sequenced on an Illumina HiSeq 4000 system, generating paired‐end 150‐bp reads, providing deep and comprehensive transcriptomic coverage.

### Single‐Nucleus ATAC‐Seq (snATAC‐Seq) Library Preparation and Sequencing

RFP^+^ pancreatic cells from E16.5 *Ngn3‐Cre^ER^; Rosa‐RFP* embryos were isolated by FACS. snATAC‐seq libraries were prepared using the Chromium Next GEM Single Cell ATAC Library and Gel Bead Kit (10× Genomics), following the manufacturer's protocol. Libraries were sequenced on the Illumina NovaSeq 6000 platform, generating 50 bp reads.

### Single‐Cell RT‒qPCR (scRT‒qPCR)

For scRT–qPCR, cDNA was synthesized individually from each isolated cell. The resulting cDNA was diluted 1:20 to optimize conditions for quantitative PCR. Quantitative PCR was performed on a Roche LightCycler 480 Instrument II. The primers used for amplification were as follows:

*Gapdh*: forward, ATGGTGAAGGTCGGTGTGAAC
    reverse, GCCTTGACTGTGCCGTTGAAT
*Ins2*: forward, TGGCTTCTTCTACACACCCA
    reverse, TCTAGTTGCAGTAGTTCTCCA
*Sst*: forward, CAACCAGACAGAGAATGATG
    reverse, AAGCTAACAGGATGTGAATG
*Gcg*: forward, TTGAGAGGCATGCTGAAGGG
    reverse, TCTTCTGGGAAGTCTCGCCT
*Ppy*: forward, CTGTTTCTCGTATCCACTTG
    reverse, GAGTTTCATATTGTGCCATCT
*Pdx1*: forward, AACTTGAGCGTTCCAATACGGA
    reverse, CAGCCGCCTTTCGTTATTCTTA

### Preprocessing of Smart‐Seq3 scRNA‐Seq Data

Paired‐end reads were first trimmed using Trimmomatic (version 0.39)^[^ ^34^ ^]^ to remove index, primer, and adapter sequences, retaining only biologically relevant fragments for downstream analysis. The trimmed reads were then aligned to the *Mus musculus* reference genome (GRCm38/mm10) using HISAT2 (version 2.1.0).^[^ ^35^ ^]^ Aligned reads were assigned to genes using featureCounts (version 2.0.1).^[^ ^36^ ^]^ Gene expression levels were normalized as transcripts per million (TPM) and log‐transformed (log₂(TPM + 1)) for downstream comparisons. To avoid data distortion by extremely high‐abundance transcripts, a set of highly expressed genes (*Ins1*, *Ins2*, *Gcg*, *Sst*, *Ttr*, *Ppy*, *Iapp*, *Pyy*, and *Yam1*) was excluded from total transcript calculation. Cells with more than 4000 detected genes (TPM > 0) were retained for further analyses.

### Preprocessing of 10× Genomics scRNA‐Seq Data from P60 Mice

Raw sequencing data were aligned to the *Mus musculus* reference genome (GRCm38/mm10) using Cell Ranger (version 3.1.0, 10× Genomics) with default parameters. A unique molecular identifier (UMI) count matrix was generated for downstream analysis. Cells with fewer than 3000 detected genes or with mitochondrial UMI fractions exceeding 20% were excluded. For normalization, gene expression values were calculated as transcripts per 0.1 million (TP0.1M), defined as the UMI count per gene divided by the total UMI count in the cell, then multiplied by 100 000. The resulting values were log‐transformed using log₂(TP0.1M + 1) prior to downstream analysis.

### Feature Selection for scRNA‐Seq Data

Feature selection was performed using the Seurat R package (version 4.1.3).^[^ ^37^ ^]^ The top 2000 highly variable genes (HVGs) were identified using the *FindVariableFeatures* function. Genes associated with cell cycle regulation and those specifically expressed in non‐endocrine cell types—potentially introduced by ambient RNA contamination or index switching—were excluded. Next, a pairwise correlation *ρ*_ρ_ matrix of the retained HVGs was constructed. Further gene filtering was applied based on the following criteria: genes must be coexpressed with at least *n* other HVGs (*ρ*_ρ_ > *c*), and expressed in a minimum min and maximum *max* fraction of total cells (default parameters: *n *= 10, *c *= 0.25, *min *= 0.01, *max* = 0.95).

### Cell Type Definition for scRNA‐Seq Data

Cell type identification was performed using the Seurat R package (version 4.1.3).^[^ ^37^ ^]^ For both the Smart‐seq3 and 10× Genomics scRNA‐seq datasets, feature selection was conducted using default parameters. The selected genes were scaled and centered using the *ScaleData* function, followed by principal component analysis (PCA) via the *RunPCA* function. Batch effects in the 10× Genomics datasets were corrected using the *fastMNN* function from the batchelor R package (version 1.8.1).^[^ ^38^ ^]^

Dimensionality reduction was conducted using UMAP (*RunUMAP*), and unsupervised clustering was performed using the *FindNeighbors* and *FindClusters* functions (Louvain algorithm). Resulting clusters were annotated based on the expression patterns of canonical endocrine markers.

Cells expressing *Spi1* and *Kdr* were identified as immune cells and mesenchymal cells, respectively. Endocrine cells expressing *Ins1*/*Ins2*, *Sst*, *Gcg*, or *Ppy* above dataset‐specific quantile thresholds were classified as *Ins*⁺ (β‐cells), *Sst*⁺ (δ‐cells), *Gcg*⁺ (α‐cells), or *Ppy*⁺ (PP‐cells), respectively.

### Bihormonal Cell Definition for scRNA‐Seq Analysis

Bihormonal cells were defined as cells co‐expressing any two of the following endocrine markers: *Ins1*/*Ins2*, *Sst*, *Gcg*, or *Ppy*. To distinguish true expression from background noise, dataset‐specific gene expression cutoffs were determined using quantile thresholds. In the mouse embryonic 10× Genomics dataset, the cutoffs for *Ins1*/*Ins2*, *Sst*, and *Gcg* were set at the 0.1 quantile, while *Ppy* was set at 0.25 (Figure S6, Supporting Information). In the adult mouse dataset, more dataset‐specific thresholds were applied: 0.55 for *Ins1*, 0.03 for *Ins2*, 0.05 for *Sst*, 0.3 for *Gcg*, and 0.07 for *Ppy* (Figure S6, Supporting Information). For human embryonic pancreas data, cutoffs were set at 0.03 for *INS* and *GCG*, and 0.001 for *SST* (Figure 4; Figure S6, Supporting Information). In the adult human dataset, *INS*, *SST*, and *GCG* were thresholded at 0.1, while *PPY* was thresholded at 0.35 (Figures S7,S8, Supporting Information). Similarly, in the hESC‐derived endocrine cell dataset, cutoffs were set at 0.1 for *INS* and *GCG*, and 0.35 for *SST* (Figure 4).

In contrast, for Smart‐seq3‐based scRNA‐seq datasets, bihormonal cells were identified by dual fluorescence labeling, which allowed direct FACS‐based isolation of cells co‐expressing two specific hormones at the protein level. This approach provided a high‐confidence definition of bihormonal identity and was applied in both embryonic and postnatal mouse tissues (Figures 3, 4).

### Identification and Removal of Doublet Cells

For Smart‐seq3 data (Figures 3, 4), artificial doublets were generated by combining one Gcg‐GFP⁺ cell and one Ppy‐mNeptune⁺ cell in the same well. PCA and transcriptomic comparisons showed that these artificial doublets formed a distinct cluster and retained mixed gene signatures, clearly separable from authentic bihormonal cells. This analysis confirmed the biological validity of the bihormonal cells identified in the dataset (Figure 3A–C; Figure S2B, Supporting Information).

For publicly available datasets used in Figure 4G–K, pre‐processed data were utilized that had undergone rigorous quality control and doublet exclusion.

For 10× Genomics data (Figure 6), given the known limitations of droplet‐based platforms in resolving doublets, all cells co‐expressing multiple hormones were conservatively excluded from downstream analyses to ensure classification accuracy (see Section 2.8).

### Gene Coexpression Network (GCN) Analysis

Following feature selection, a pairwise gene–gene correlation (*ρ*_ρ_) matrix was constructed. This matrix was used to generate an undirected weighted graph using the *graph.adjacency* function from the igraph R package (version 1.4.1, https://cran.r‐project.org/src/contrib/Archive/igraph/igraph_1.4.1.tar.gz). In this graph, each highly coexpressed gene was represented as a vertex, and edges were drawn between gene pairs with *ρ*_ρ_ values exceeding a defined threshold. Gene modules within this network were identified using the *cluster_walktrap* function, which is available in the igraph R package. Hierarchical clustering was subsequently applied to these gene modules to delineate endocrine cell subtypes based on their coexpressed gene profiles.

### Differentially Expressed Gene (DEG) Analysis

DEGs were identified using the *FindAllMarkers* function in the Seurat R package (version 4.1.3), with the Wilcoxon rank‐sum test specified by the parameter *test.use = “wilcox”*.

### Similarity Analysis of Gcg^+^Ppy^+^ Bihormonal Cells

To assess the similarity between *Gcg* ^+^ *Ppy* ^+^ bihormonal cells and α‐ or PP‐cells, the following procedures were conducted. First, DEGs between α‐ and PP‐cells were identified with the parameters p‐adj ≤ 0.05 and log_2_(fold change) ≥ 0.5. Then, a pseudobulk matrix of DEGs was created by calculating the average gene expression levels of these DEGs in α‐ and PP‐cells. Subsequently, the similarity score was quantified by computing the Spearman correlation coefficient of the DEGs between each individual single cell and the pseudobulk α‐ or PP‐cells. Cells exhibiting a higher similarity score to α‐cells than to PP‐cells were classified as α‐like *Gcg* ^+^ *Ppy* ^+^ bihormonal cells, whereas cells with a lower similarity score to α‐cells than to PP‐cells were categorized as PP‐like *Gcg* ^+^ *Ppy* ^+^ bihormonal cells.

### Gene Regulatory Network Analysis

Gene regulatory networks analysis was conducted using the SCENIC R package (version 1.3.0)^[^ ^39^ ^]^ with default parameters. The mean expression level of putative target genes within each regulon was used to represent the overall regulon activity. A network plot was generated to visualize the top 25 downstream target genes, providing an overview of the key regulatory interactions.

### Gene Ortholog Mapping

Orthologous gene pairs between human and mouse were obtained using the biomaRt R package (version 2.48.3).^[^ ^40^ ^]^ To enable cross‐species comparisons, Ensembl gene names from human datasets were converted to their corresponding mouse Ensembl gene names.

### 10x Genomics snATAC‐Seq Analysis for Mouse and Human α‐, β‐ and δ‐Cells

For both mouse and human datasets, snATAC‐seq data were processed using a unified pipeline incorporating the R packages Seurat (version 4.1.3)^[^ ^37^ ^]^ and Signac (version 1.3.0).^[^ ^41^ ^]^ The sequencing data were aligned against the appropriate reference genomes (refdata‐celranger‐atac‐mm10‐1.2.0 for mouse) using cellranger‐atac (version 1.2.0) with standard settings. Peak calling was conducted using macs2 (version 2.2.6),^[^ ^42^ ^]^ with exclusion of regions overlapping ENCODE blacklisted genomic regions. The genomic regions in humans were mapped and annotated with the human genome EnsDb.Hsapiens.v86 (version 2.99.0) and BSgenome.Hsapiens.UCSC.hg38 (version 2.99.0). Cells exhibiting quality metrics, such as UMI counts surpassing 1000 and specific read thresholds, were retained for further analysis. In particular, mouse cells were filtered based on reads in promoter areas (RIPRs) ranging from 20% to 50%, while human δ‐cells were selected with read counts between 5000 and 50 000 and nucleosome signals below 2.

The snATAC‐seq datasets were normalized using the *RunTFIDF* and *RunSVD* functions from Signac, and the most variable features (HVFs) were identified using the *FindTopFeatures* function. Batch effects were mitigated using the *reducedMNN* function from the R package batchelor (version 1.8.1).

For human δ‐cells, to build a joint neighbor graph, the *FindMultiModalNeighbors* function from Seurat was employed, based on “pca” reduction for snRNA‐seq data and “integratedLSI” reduction for snATAC‐seq data. Cell identities were assigned based on chromatin accessibility signatures at lineage‐specific regions, such as *Gcg* for α‐cells and *Ins1, Ins2* for β‐cells. Promoter accessibility for both human and mouse datasets was further calculated using the *GeneActivity* function from Signac. For human δ‐cells, differential motif accessibility analysis was performed using the *RunChromVAR* function^[^ ^43^ ^]^ leveraging motif position frequency matrices from the R package JASPAR2020 (version 0.99.10).^[^ ^44^ ^]^ Differentially accessible peaks were identified using the *FindAllMarkers* function from Seurat with the LR test.

For specific chromatin regions (e.g., *Arx, Mnx1, Gcg, Ins1*, and *Ins2*), individual cell signal intensities were measured, and cells were ranked by these signal intensities. Mouse α‐cells exhibiting more than 45% and 47% of the highest signal intensities in β‐cells at the *Ins1* and *Ins2* regions, respectively, were categorized as having high accessibility.

## Conflict of Interest

The authors declare no conflict of interest.

## Author Contributions

C.‐R.X. conceived the project. C.‐R.X., X.‐X.Y., and M.‐Y.H. designed the research. X.‐X.Y., P.P., M.‐Y.H., L.Y., X.W., and J.‐X.Z. performed the research. X.‐X.Y., Y.‐N.W., C.‐T.J., M.‐Y.H., S.H., J.G., and C.‐R.X. analyzed the data. X.‐X.Y., M.‐Y.H., S.H., and C.‐R.X. wrote the manuscript.

## Supporting information



Supporting Information

Supplemental TableS1

Supplemental TableS2

Supplemental TableS3

Supplemental TableS4

Supplemental TableS5

Supplemental TableS6

## Data Availability

The RNA‐seq data from this study have been deposited in the Gene Expression Omnibus (GEO) database and assigned the identifier GSE248196. The following link was created to permit the review of records while ensuring that the records remained private: https://www.ncbi.nlm.nih.gov/geo/query/acc.cgi?acc=GSE248196. (secure token: yhuxiqowfrsvxgh). Code used in this paper is available from the lead contact upon request.
