# Supplementary material for: Comprehensive Characterization of Bihormonal Cells and Endocrine Cell Lineages in Mammalian Pancreatic Islets
Source: Adv Sci (Weinh). 2025 May 29;12(30):e16326. doi: 10.1002/advs.202416326 (PMC12376548; doi:10.1002/advs.202416326)
Supplement: Supplementary file 1 — Supporting Information [file ADVS-12-e16326-s003.pdf]

ADVANCED SCIENCE
Open Access

Supporting Information

for *Adv. Sci.*, DOI 10.1002/advs.202416326

Comprehensive Characterization of Bihormonal Cells and Endocrine Cell Lineages in Mammalian Pancreatic Islets

*Xin-Xin Yu, Peng Peng, Yi-Ning Wang, Mao-Yang He, Shuang He, Chen-Tao Jin, Liu Yang, Xi Wang, Jia-Xi Zheng, Jie Gao and Cheng-Ran Xu**

Supplementary Information

Comprehensive Characterization of Bihormonal Cells and Endocrine Cell Lineages in Mammalian Pancreatic Islets

Xin-Xin Yu[1,3], Peng Peng[1,3], Yi-Ning Wang[1,3], Mao-Yang He[1,2,3,4], Shuang He[3,4], Chen-Tao Jin[1,3], Liu Yang[1,3], Xi Wang[1,3], Jia-Xi Zheng[5], Jie Gao[5] and Cheng-Ran Xu[1,3*]

[1] *State Key Laboratory of Female Fertility Promotion, Department of Medical Genetics, School of Basic Medical Sciences, Peking University, Beijing 100191, China*

[2] *PKU-Tsinghua-NIBS Graduate Program, Peking University, Beijing 100871, China*

[3] *Peking-Tsinghua Center for Life Sciences, Peking University, Beijing 100871, China*

[4] *School of Life Sciences, Peking University, Beijing, 100871, China*

[5] *Department of Hepatobiliary Surgery, Peking University People's Hospital, Beijing, 100044, China*

[*] Correspondence: Cheng-Ran Xu, Ph.D.

cxu@pku.edu.cn

This PDF file includes:

Figures S1 to S9

Other Supplemental Materials for this manuscript include:

Tables S1 to S6

Figure S1

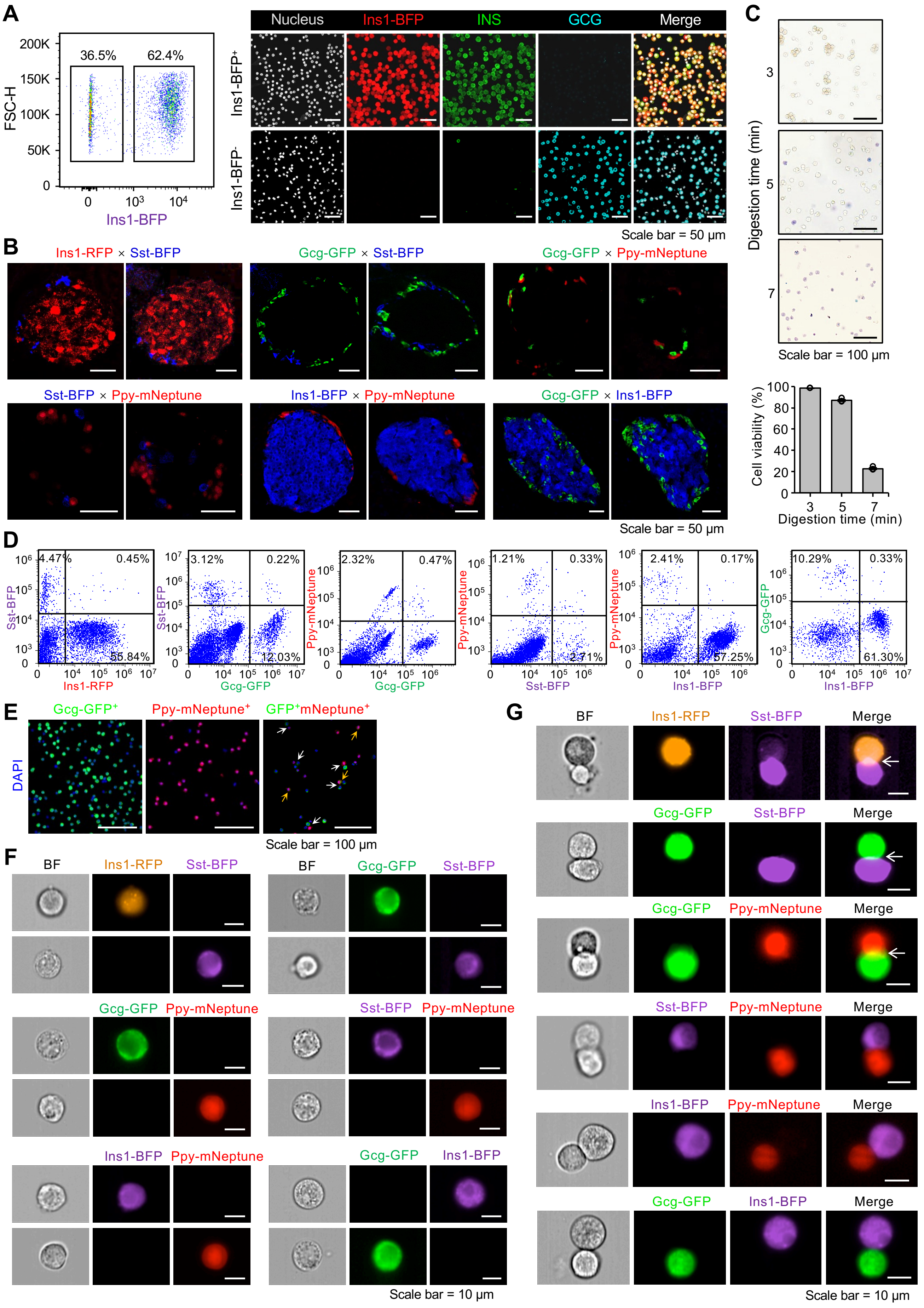

A
B
C
D
E
F
G
Nucleus
Ins1-BFP
INS
GCG
Merge
Ins1-BFP+
Ins1-BFP-
FSC-H
Scale bar = 50 μm
Ins1-RFP × Sst-BFP
Gcg-GFP × Sst-BFP
Gcg-GFP × Ppy-mNeptune
Sst-BFP × Ppy-mNeptune
Ins1-BFP × Ppy-mNeptune
Gcg-GFP × Ins1-BFP
Digestion time (min)
Scale bar = 100 μm
Cell viability (%)
Gcg-GFP+
Ppy-mNeptune+
GFP+mNeptune+
DAPI
BF
Ins1-RFP
Sst-BFP
Gcg-GFP
Ppy-mNeptune
Scale bar = 10 μm

Figure S1. Optimization of cell enrichment and validation of reporter specificity for mouse pancreatic endocrine cells.

A) Validation of the Ins1-BFP transgenic reporter. Left: flow cytometry analysis showing the proportion of BFP^{+} and BFP^{-} cells in *Ins1-BFP* mice. Right: Immunofluorescence staining of sorted cells with antibodies against insulin (INS, green) and glucagon (GCG, cyan). BFP fluorescence (red) overlaps extensively with INS^{+} cells but not with GCG^{+} cells. Nuclei were counterstained with SYTOX™ Deep Red (white). The merged image confirms high specificity of *Ins1-BFP* labeling. Scale bars, 50 µm.

B) Representative images of frozen sections from six different dual-reporter P60 mouse islets. Scale bars, 50 µm.

C) Assessment of cell integrity under different enzymatic digestion times. Top: Trypan blue staining of dead cells. Bottom: quantification of cell viability (%) across digestion times. Data are presented as mean ± SEM (n = 3 mice per group). Scale bars, 100 µm.

D) FACS gating strategies used to isolate six types of dual-fluorescent endocrine cells from adult mouse islets.

E) Confocal images of sorted cells showing Gcg-GFP^{+} single-positive, Ppy-$mNeptune^{+}$ single-positive, and $GFP^{+}mNeptune^{+}$ double-positive populations. White arrows indicate cell doublets; yellow arrows indicate bona fide bihormonal cells. Scale bars, 100 µm.

F) IFC images of cells from single-positive fluorescent channels. Scale bars, 10 µm.

G) IFC images showing doublets from six dual-labeled islet combinations. White arrows indicate regions of overlapping hormone signals. Scale bars, 10 µm.

Figure S2

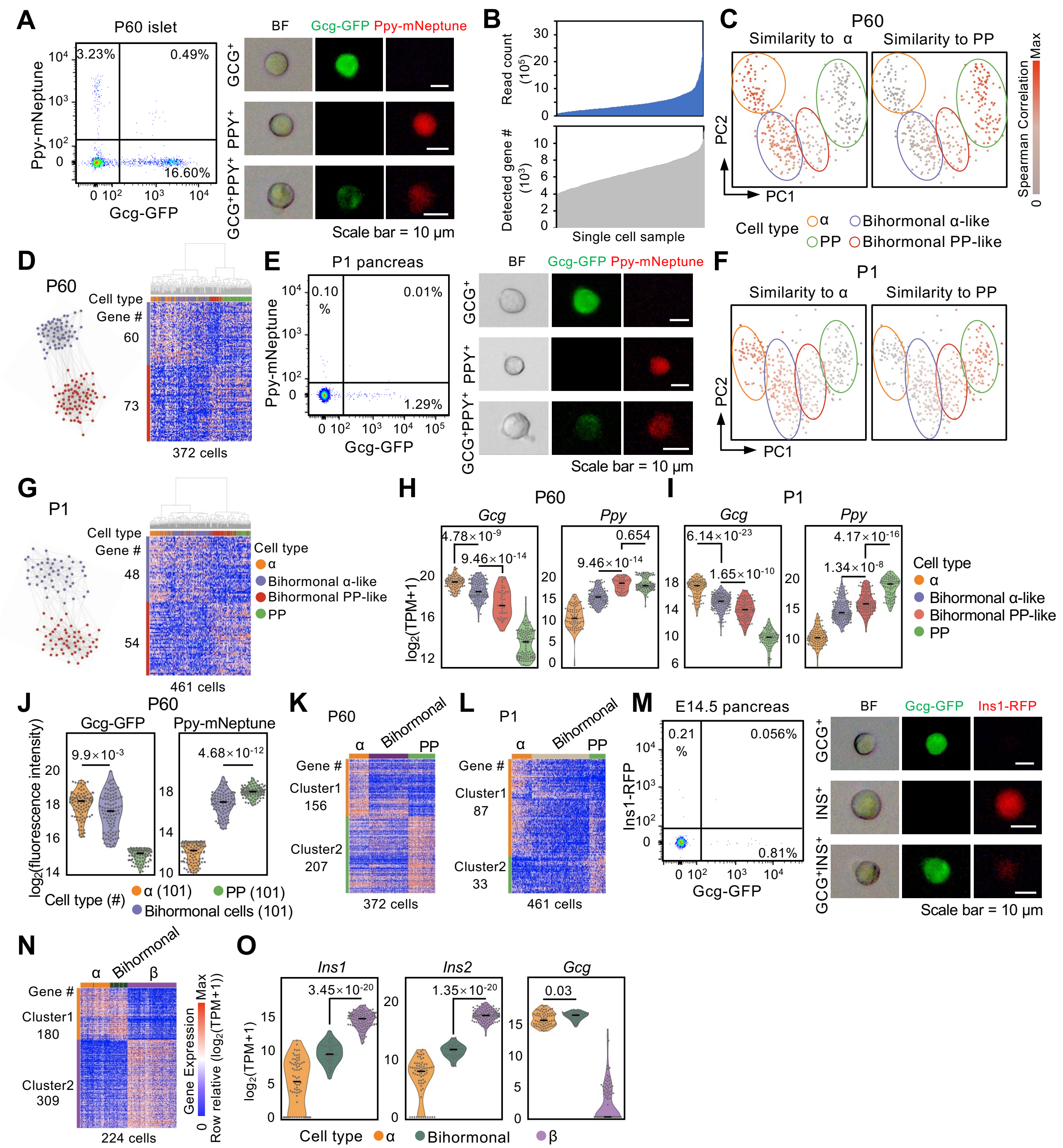
A
P60 islet
Ppy-mNeptune
Gcg-GFP
3.23%
0.49%
16.60%
BF
Gcg-GFP Ppy-mNeptune
GCG⁺
PPY⁺
GCG⁺PPY⁺
Scale bar = 10 μm
B
Read count (10^5)
Detected gene # (10^3)
Single cell sample
C
P60
Similarity to α
Similarity to PP
PC1
PC2
Spearman Correlation
Max
0
Cell type
α
PP
Bihormonal α-like
Bihormonal PP-like
D
P60
Cell type
Gene #
60
73
372 cells
E
P1 pancreas
0.10 %
0.01%
1.29%
Ppy-mNeptune
Gcg-GFP
BF
Gcg-GFP Ppy-mNeptune
GCG⁺
PPY⁺
GCG⁺PPY⁺
Scale bar = 10 μm
F
P1
Similarity to α
Similarity to PP
PC1
PC2
G
P1
Cell type
Gene #
48
54
461 cells
Cell type
α
Bihormonal α-like
Bihormonal PP-like
PP
H
P60
Gcg
Ppy
4.78×10^{-9}
9.46×10^{-14}
0.654
9.46×10^{-14}
$\log_2(\mathrm{TPM}+1)$
I
P1
Gcg
Ppy
6.14×10^{-23}
1.65×10^{-10}
4.17×10^{-16}
1.34×10^{-8}
Cell type
α
Bihormonal α-like
Bihormonal PP-like
PP
J
P60
Gcg-GFP
Ppy-mNeptune
9.9×10^{-3}
4.68×10^{-12}
$\log_2$(fluorescence intensity)
Cell type (#)
α (101)
PP (101)
Bihormonal cells (101)
K
P60
α
Bihormonal
PP
Gene #
Cluster1
156
Cluster2
207
372 cells
L
P1
α
Bihormonal
PP
Gene #
Cluster1
87
Cluster2
33
461 cells
M
E14.5 pancreas
0.21 %
0.056%
0.81%
Ins1-RFP
Gcg-GFP
BF
Gcg-GFP
Ins1-RFP
GCG⁺
INS⁺
GCG⁺INS⁺
Scale bar = 10 μm
N
α
Bihormonal
β
Gene #
Cluster1
180
Cluster2
309
224 cells
Max
Gene Expression
Row relative ($\log_2(\mathrm{TPM}+1)$)
0
O
Ins1
Ins2
Gcg
3.45×10^{-20}
1.35×10^{-20}
0.03
$\log_2(\mathrm{TPM}+1)$
Cell type
α
Bihormonal
β

Figure S2. Enrichment and transcriptomic characterization of Gcg-GFP$^+$Ppy-mNeptune$^+$ and Gcg-GFP$^+$Ins1-RFP$^+$ cells for scRNA-seq analysis.

A) FACS gating strategy for isolating Gcg-GFP$^+$Ppy-mNeptune$^+$ cells from P60 mouse pancreata (left) and single-cell imaging verification (right). Scale bars, 10 µm.

B) Distribution of sequencing read counts and detected genes per cell from Smart-seq3 scRNA-seq data. Each bar represents an individual cell.

C) PCA plots showing the similarity of P60 α-cells and PP-cells to each analyzed Gcg$^+$Ppy$^+$ cell. Similarity scores were calculated using Spearman correlation.

D, G) GCN and hierarchical clustering analysis of α-like and PP-like *Gcg*$^+$*Ppy*$^+$ cells from P60 (D) and P1 (G) mice. Gene counts per cluster are annotated. Each column represents a cell; each row represents a gene.

E) FACS gating strategy (left) and microscopic validation (right) of Gcg-GFP$^+$Ppy-mNeptune$^+$ cells from P1 mouse pancreata. Scale bars, 10 µm.

F) PCA plots showing Spearman correlation–based similarity scores of Gcg$^+$Ppy$^+$ cells to P1 α-cells and PP-cells.

H, I) Violin plots of Gcg and *Ppy* gene expression in cells from P60 (H) and P1 (I) pancreata. Black lines indicate median expression levels. Statistical comparisons were performed using the unpaired Wilcoxon rank-sum test.

J) Violin plots of GFP and mNeptune fluorescence intensities in P60 islet cells based on IFC data. Each dot represents a single cell; cell counts are indicated in brackets. Median intensity is marked by a black line. Statistical comparisons were performed using the unpaired Wilcoxon rank-sum test.

K, L) Heatmaps of DEGs among α-cells, PP-cells, and *Gcg*$^+$*Ppy*$^+$ cells in P60 (K) and P1 (L) samples. Each column represents a single cell; each row a gene. DEGs were identified by unpaired Wilcoxon rank-sum test using the following thresholds: p-adj ≤ 0.05, $\log_2$(fold change) ≥ 0.5 (K) or ≥ 0.8 (L), pct.1 ≥ 0.5, pct.2 ≤ 0.9 (K) or ≤ 0.5 (L).

M) FACS gating strategy (left) and imaging verification (right) of Gcg-GFP$^+$Ins1-RFP$^+$ cells isolated from E14.5 mouse embryonic pancreas. Scale bars, 10 µm.

N) Heatmap of DEGs among α-cells, β-cells, and *Gcg*$^+$*Ins*$^+$ bihormonal cells at E14.5. Unpaired Wilcoxon rank-sum test, p-adj ≤ 0.05, $\log_2$(fold change) ≥ 0.5, pct.1 ≥ 0.5, and pct.2 ≤ 0.9.

O) Violin plots of *Ins1*, *Ins2*, and *Gcg* gene expression in in single cells from E14.5 embryos. Statistical analysis was performed using the unpaired Wilcoxon rank-sum test.

Figure S3

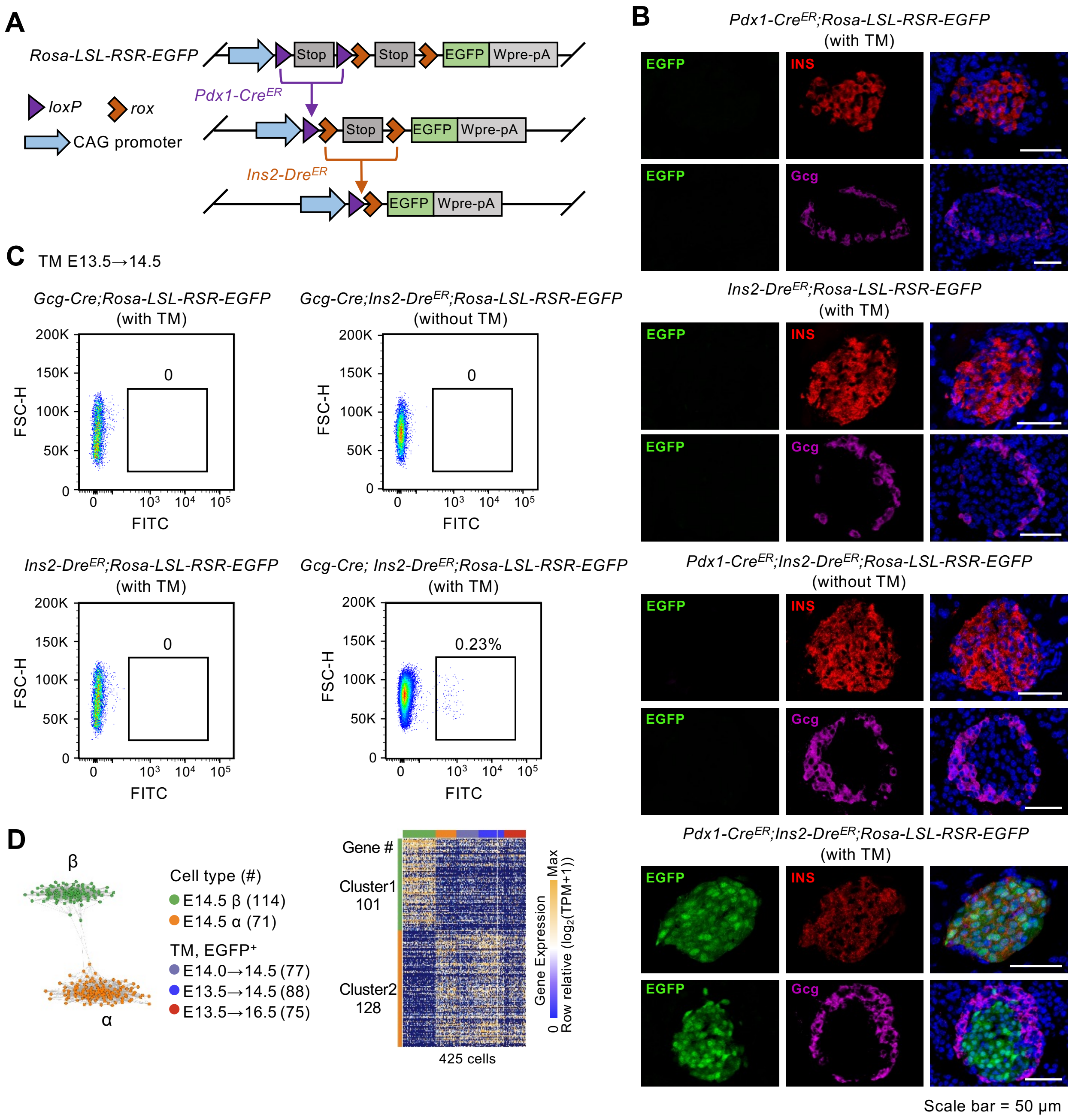

A
Rosa-LSL-RSR-EGFP
Stop
Stop
EGFP
Wpre-pA
loxP
rox
CAG promoter
Pdx1-CreER
Ins2-DreER
B
Pdx1-CreER;Rosa-LSL-RSR-EGFP (with TM)
Ins2-DreER;Rosa-LSL-RSR-EGFP (with TM)
Pdx1-CreER;Ins2-DreER;Rosa-LSL-RSR-EGFP (without TM)
Pdx1-CreER;Ins2-DreER;Rosa-LSL-RSR-EGFP (with TM)
EGFP
INS
Gcg
Scale bar = 50 μm
C
TM E13.5→14.5
Gcg-Cre;Rosa-LSL-RSR-EGFP (with TM)
Gcg-Cre;Ins2-DreER;Rosa-LSL-RSR-EGFP (without TM)
Ins2-DreER;Rosa-LSL-RSR-EGFP (with TM)
Gcg-Cre; Ins2-DreER;Rosa-LSL-RSR-EGFP (with TM)
FSC-H
FITC
0
0.23%
D
β
α
Cell type (#)
E14.5 β (114)
E14.5 α (71)
TM, EGFP+
E14.0→14.5 (77)
E13.5→14.5 (88)
E13.5→16.5 (75)
Gene #
Cluster1 101
Cluster2 128
425 cells
Gene Expression
Row relative (log2(TPM+1))
0
Max

Figure S3. Validation of the dual-recombinase system for cell labeling in mice.

A) Schematic illustration of the dual-recombinase strategy for β-cell labeling using the *Pdx1-Cre*^{ER}*;Ins2-Dre*^{ER}*;Rosa-LSL-RSR-EGFP* mouse model.

B) Immunofluorescence staining for EGFP, INS, and GCG in frozen pancreatic sections from from the following genotypes: *Pdx1-Cre*^{ER}*;Rosa-LSL-RSR-EGFP*, *Ins2-Dre*^{ER}*;Rosa-LSL-RSR-EGFP*, and *Pdx1-Cre*^{ER};*Ins2-Dre*^{ER}*;Rosa-LSL-RSR-EGFP*, with or without tamoxifen (TM) treatment. Scale bars, 50 μm.

C) FACS gating strategy for isolating EGFP^{+} cells from *Gcg-Cre;Ins2-Dre*^{ER}*;Rosa-LSL-RSR-EGFP* mice following TM administration at E13.5 and analysis at E14.5 (TM E13.5→14.5).

D) GCN and heatmap analysis of α-cells, β-cells, and EGFP^{+} cells isolated from dual-recombinase–based lineage tracing.

Figure S4

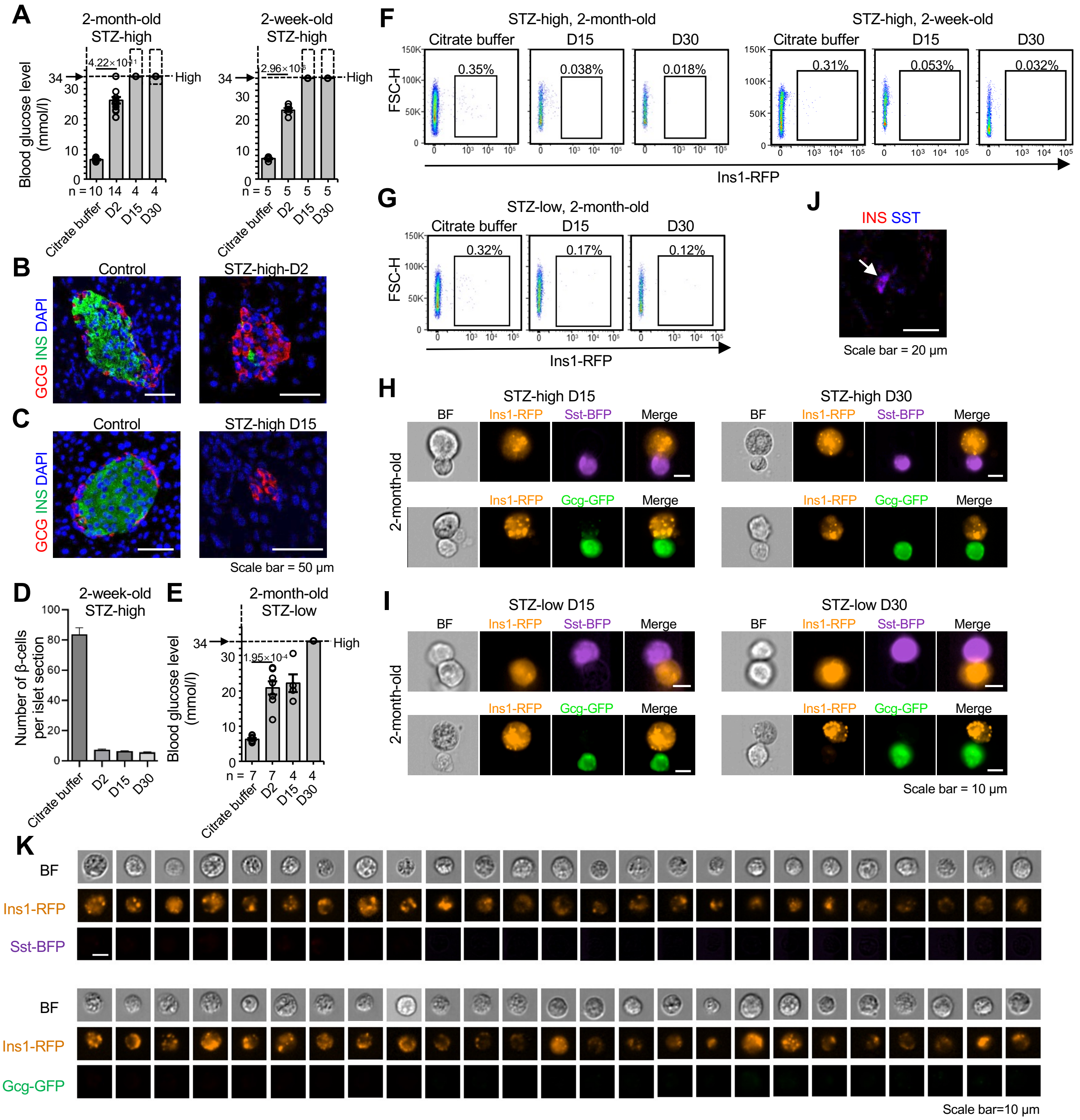

A
2-month-old STZ-high
2-week-old STZ-high
Blood glucose level (mmol/l)
4.22×10⁻¹¹
2.96×10⁻⁶
34
High
n = 10 14 4 4
n = 5 5 5 5
Citrate buffer
D2
D15
D30
B
Control
STZ-high-D2
GCG INS DAPI
C
Control
STZ-high D15
Scale bar = 50 μm
D
2-week-old STZ-high
Number of β-cells per islet section
E
2-month-old STZ-low
1.95×10⁻⁴
n = 7 7 4 4
F
STZ-high, 2-month-old
STZ-high, 2-week-old
Citrate buffer
0.35%
0.038%
0.018%
0.31%
0.053%
0.032%
FSC-H
Ins1-RFP
G
STZ-low, 2-month-old
0.32%
0.17%
0.12%
J
INS SST
Scale bar = 20 μm
H
STZ-high D15
STZ-high D30
BF
Ins1-RFP
Sst-BFP
Gcg-GFP
Merge
2-month-old
I
STZ-low D15
STZ-low D30
Scale bar = 10 μm
K
BF
Ins1-RFP
Sst-BFP
Gcg-GFP
Scale bar=10 μm

Figure S4. Analysis of bihormonal cells in the pancreas of STZ-treated mice.

A) Nonfasting blood glucose levels in 2-month-old (left) or 2-week-old (right) mice at various time points after high-dose STZ (STZ-high) or citrate buffer control administration. The dotted line indicates the upper detection limit of the glucose meter (34 mmol/L). Data are presented as mean ± SEM. Statistical analysis was performed using Welch's t test. n indicates the number of mice per group.

B, C) Representative immunofluorescence images of pancreatic sections from D2 (B) and D15 (C) mice after citrate buffer or STZ-high administration at 2-week-old. Scale bars, 50 μm.

D) Quantification of residual β-cells per islet section at different time points following STZ-high treatment in 2-week-old mice.

E) Non-fasting blood glucose levels in 2-month-old mice at indicated time points following low-dose STZ (STZ-low) or citrate buffer administration. Data are shown as mean ± SEM. Welch's t test was used for statistical comparison.

F, G) FACS analysis of whole pancreata from 2-month-old (left) and 2-week-old (right) mice after high-dose STZ treatment (F), and from 2-month-old mice after low-dose STZ treatment (G) at different time points.

H, I) IFC images showing representative cell doublets from 2-month-old mice at various time points after STZ-high (H) or STZ-low (I) treatment. Scale bars, 10 μm.

J) Immunofluorescent staining for INS and SST in pancreatic tissues from the D15 post-STZ-high treatment at 2-week-old. The arrow indicates an INS^+SST^+ bihormonal cell. Scale bar, 20 μm.

K) IFC images of Ins1-RFP^+ cells in pancreata from *Ins1-RFP;Sst-BFP* and *Gcg-GFP;Ins1-RFP* mice on 4 months post STZ-high treatment (administered at 2 weeks of age). Scale bars, 10 μm.

Figure S5

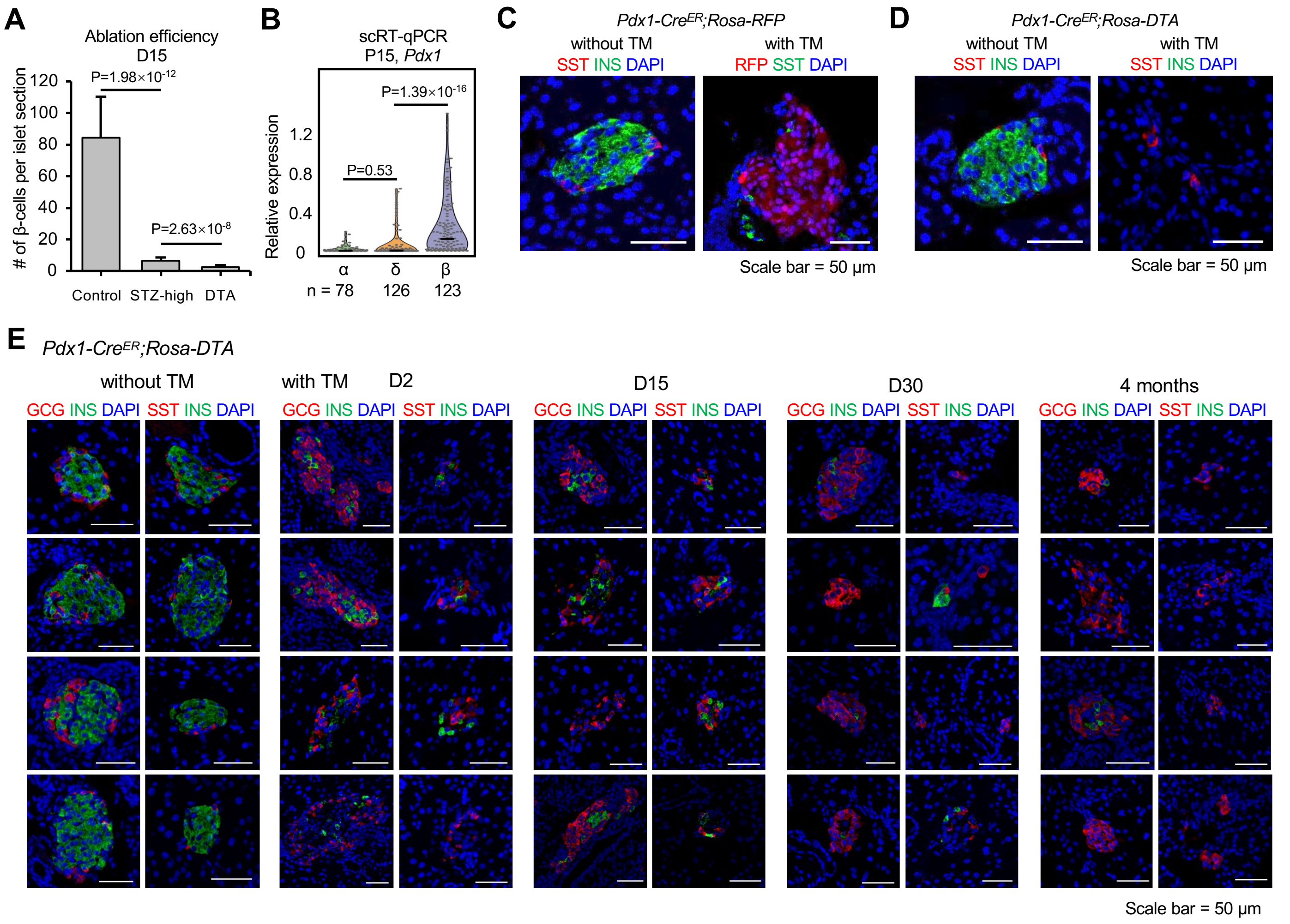
A
Ablation efficiency
D15
# of β-cells per islet section
P=1.98×10-12
P=2.63×10-8
Control
STZ-high
DTA
B
scRT-qPCR
P15, Pdx1
Relative expression
P=0.53
P=1.39×10-16
α
δ
β
n = 78
126
123
C
Pdx1-CreER;Rosa-RFP
without TM
SST INS DAPI
with TM
RFP SST DAPI
Scale bar = 50 μm
D
Pdx1-CreER;Rosa-DTA
without TM
SST INS DAPI
with TM
SST INS DAPI
Scale bar = 50 μm
E
Pdx1-CreER;Rosa-DTA
without TM
with TM
D2
D15
D30
4 months
GCG INS DAPI
SST INS DAPI
Scale bar = 50 μm

Figure S5. Analysis of bihormonal cells in the pancreas of *Pdx1-Cre^{ER};Rosa-DTA* mice following tamoxifen treatment.

A) Quantification of residual β-cells per islet section at day 15 (D15) following citrate buffer (control), STZ-high, or DTA treatment in P15 mice. Data are presented as mean ± SD. Statistical analysis was performed using Student's t test.

B) Box plots showing *Pdx1* expression levels in single cells obtained by scRT-qPCR from P15 α-cells, δ-cells, and β-cells. Median values are indicated by black lines. The unpaired Wilcoxon rank-sum test was used for statistical comparison. n indicates the number of islet sections analyzed.

C) Representative immunofluorescence staining of pancreatic sections from *Pdx1-Cre^{ER};Rosa-RFP* mice without (left) or with (right) tamoxifen (TM) treatment at P15, examined two days later. Scale bars, 50 µm.

D) Representative immunofluorescence images of *Pdx1-Cre^{ER};Rosa-DTA* mouse pancreata without (left) or with (right) TM treatment at P15, collected two days post-injection. Scale bars, 50 µm.

E) Time course of representative immunofluorescence staining in *Pdx1-Cre^{ER};Rosa-DTA* mice treated with TM at P15 and analyzed at day 2 (D2), D15, D30, and 4 months post-treatment. Scale bars, 50 µm.

Figure S6

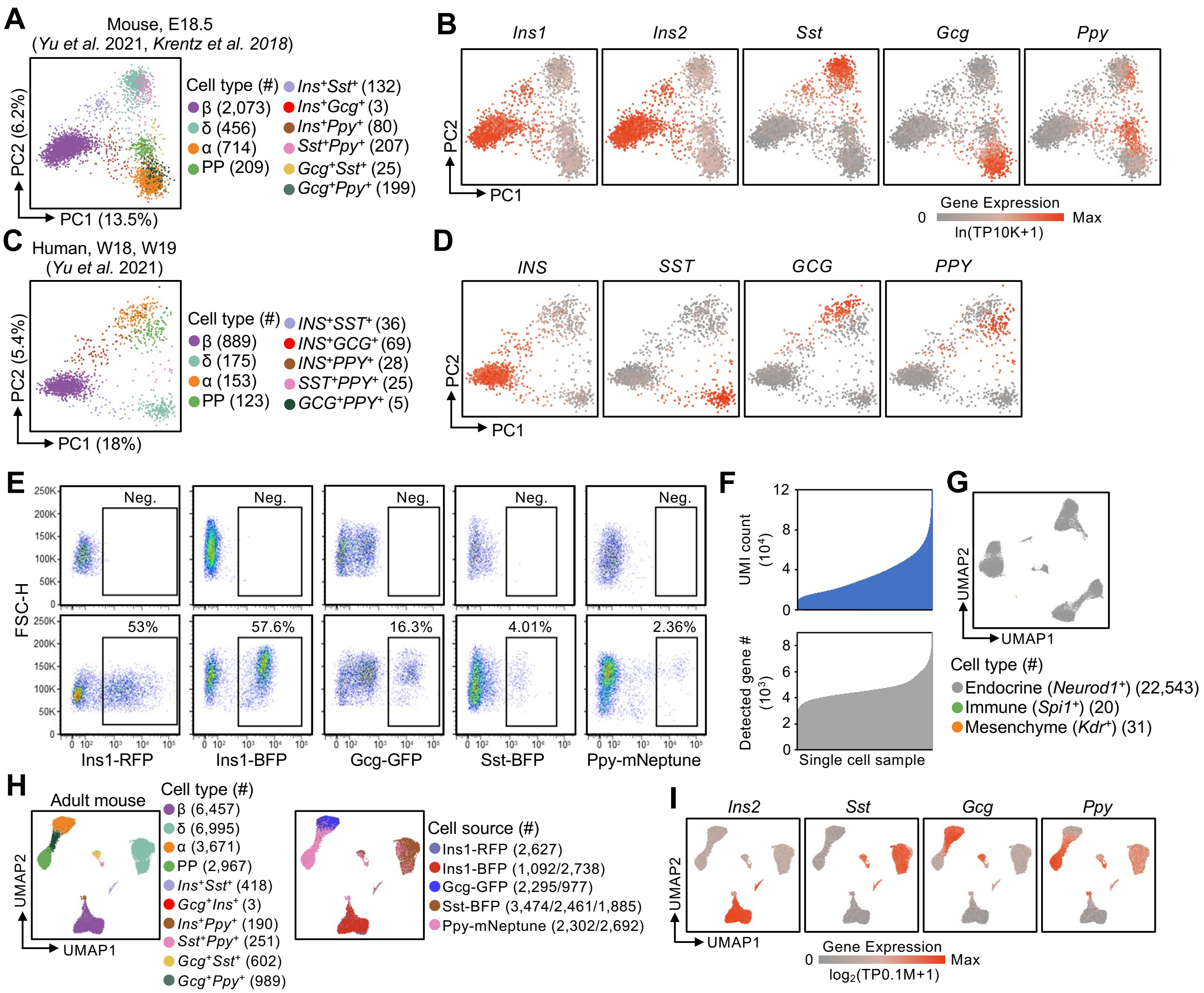

A
Mouse, E18.5
(Yu et al. 2021, Krentz et al. 2018)
PC1 (13.5%)
PC2 (6.2%)
Cell type (#)
β (2,073)
δ (456)
α (714)
PP (209)
Ins+Sst+ (132)
Ins+Gcg+ (3)
Ins+Ppy+ (80)
Sst+Ppy+ (207)
Gcg+Sst+ (25)
Gcg+Ppy+ (199)
B
Ins1
Ins2
Sst
Gcg
Ppy
PC1
PC2
Gene Expression
0
Max
ln(TP10K+1)
C
Human, W18, W19
(Yu et al. 2021)
PC1 (18%)
PC2 (5.4%)
Cell type (#)
β (889)
δ (175)
α (153)
PP (123)
INS+SST+ (36)
INS+GCG+ (69)
INS+PPY+ (28)
SST+PPY+ (25)
GCG+PPY+ (5)
D
INS
SST
GCG
PPY
PC1
PC2
E
FSC-H
Neg.
53%
57.6%
16.3%
4.01%
2.36%
Ins1-RFP
Ins1-BFP
Gcg-GFP
Sst-BFP
Ppy-mNeptune
F
UMI count (10^4)
Detected gene # (10^3)
Single cell sample
G
UMAP1
UMAP2
Cell type (#)
Endocrine (Neurod1+) (22,543)
Immune (Spi1+) (20)
Mesenchyme (Kdr+) (31)
H
Adult mouse
UMAP1
UMAP2
Cell type (#)
β (6,457)
δ (6,995)
α (3,671)
PP (2,967)
Ins+Sst+ (418)
Gcg+Ins+ (3)
Ins+Ppy+ (190)
Sst+Ppy+ (251)
Gcg+Sst+ (602)
Gcg+Ppy+ (989)
Cell source (#)
Ins1-RFP (2,627)
Ins1-BFP (1,092/2,738)
Gcg-GFP (2,295/977)
Sst-BFP (3,474/2,461/1,885)
Ppy-mNeptune (2,302/2,692)
I
Ins2
Sst
Gcg
Ppy
UMAP1
UMAP2
Gene Expression
0
Max
log2(TP0.1M+1)

Figure S6. Comparative analysis of embryonic and adult pancreatic endocrine cells in mouse and human.

A) PCA plot of E18.5 mouse endocrine cells based on scRNA-seq data from Yu et al.[1a] and Krentz et al.[25] Each dot represents a single cell; cell counts are indicated in brackets.

B) PCA plots showing the expression levels of representative marker genes for each endocrine cell type identified in (A).

C) Identification of endocrine cell types at weeks 18 and 19 (W18, W19) of human embryonic pancreatic development using data from Yu et al.[1a]

D) PCA plots showing the expression of marker genes for the cell types identified in (C).

E) FACS gating strategies for isolating Ins1-RFP$^+$, Ins1-BFP$^+$, Gcg-GFP$^+$, Sst-BFP$^+$, and Ppy-mNeptune$^+$ cells from adult (P60) mouse islets. Wild-type mouse islets were used as a negative control.

F) Statistics of unique molecular identifiers (UMIs) and number of genes detected in adult mouse islets using 10x Genomics scRNA-seq.

G) UMAP plot showing cell type clusters from P60 mouse pancreatic islets. Each dot represents a single cell; cell counts are shown in brackets.

H) UMAP plots showing endocrine cell type distribution (left) and sample origin (right) in P60 mouse islets. Each dot represents a single cell; sample sizes are indicated by slashes.

I) UMAP plots showing expression levels of representative marker genes for each endocrine cell type identified in (H).

Figure S7

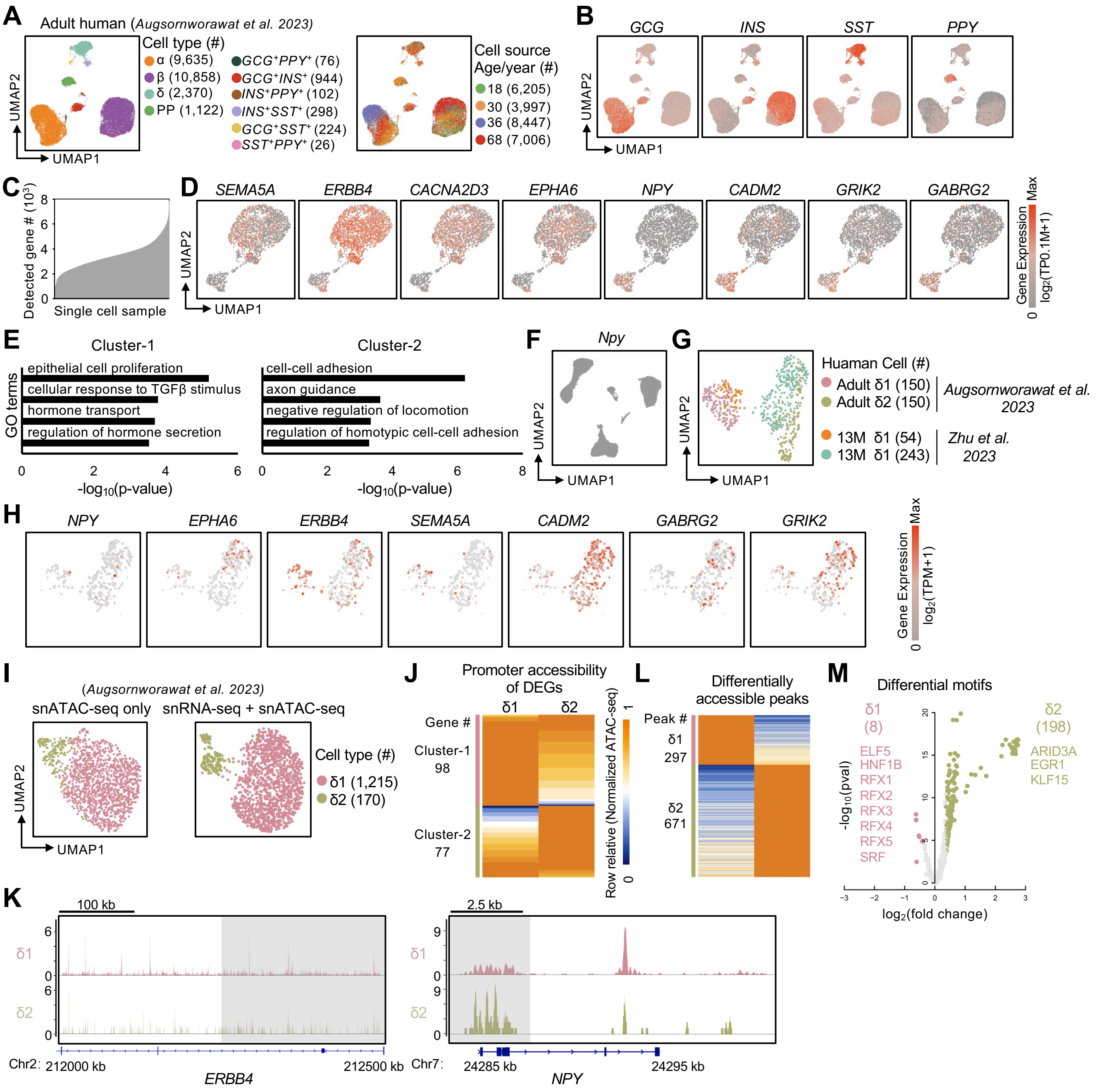
A
Adult human (Augsornworawat et al. 2023)
Cell type (#)
α (9,635)
β (10,858)
δ (2,370)
PP (1,122)
GCG+PPY+ (76)
GCG+INS+ (944)
INS+PPY+ (102)
INS+SST+ (298)
GCG+SST+ (224)
SST+PPY+ (26)
Cell source
Age/year (#)
18 (6,205)
30 (3,997)
36 (8,447)
68 (7,006)
UMAP2
UMAP1
B
GCG
INS
SST
PPY
C
Detected gene # (10^3)
Single cell sample
D
SEMA5A
ERBB4
CACNA2D3
EPHA6
NPY
CADM2
GRIK2
GABRG2
Gene Expression log2(TP0.1M+1)
Max
0
E
Cluster-1
epithelial cell proliferation
cellular response to TGFβ stimulus
hormone transport
regulation of hormone secretion
Cluster-2
cell-cell adhesion
axon guidance
negative regulation of locomotion
regulation of homotypic cell-cell adhesion
GO terms
-log10(p-value)
F
Npy
G
Huaman Cell (#)
Adult δ1 (150)
Adult δ2 (150)
Augsornworawat et al. 2023
13M δ1 (54)
13M δ1 (243)
Zhu et al. 2023
H
NPY
EPHA6
ERBB4
SEMA5A
CADM2
GABRG2
GRIK2
Gene Expression log2(TPM+1)
I
(Augsornworawat et al. 2023)
snATAC-seq only
snRNA-seq + snATAC-seq
Cell type (#)
δ1 (1,215)
δ2 (170)
J
Promoter accessibility of DEGs
δ1
δ2
Gene #
Cluster-1
98
Cluster-2
77
Row relative (Normalized ATAC-seq)
L
Differentially accessible peaks
Peak #
δ1
297
δ2
671
M
Differential motifs
δ1
(8)
ELF5
HNF1B
RFX1
RFX2
RFX3
RFX4
RFX5
SRF
δ2
(198)
ARID3A
EGR1
KLF15
-log10(pval)
log2(fold change)
K
100 kb
δ1
δ2
Chr2: 212000 kb
212500 kb
ERBB4
2.5 kb
Chr7:
24285 kb
24295 kb
NPY

Figure S7. scRNA-seq and snATAC-seq analyses of δ-cell subtypes in adult and infant human pancreas.

A) UMAP plot of adult human pancreatic endocrine cell types from the dataset by Augsornworawat et al.[27] Cell types and sample origins are color-coded.

B) UMAP plots showing expression of representative marker genes for each endocrine cell type identified in (A).

C) Statistics of of gene counts per cell in the adult human pancreas dataset from Augsornworawat et al.[27]

D) UMAP plots showing expression of genes distinguishing δ1- and δ2-cell populations, as referenced in Figure 6P.

E) Selected Gene Ontology (GO) terms enriched in cluster 1 (δ1-cells) and cluster 2 (δ2-cells).

F) UMAP plot showing *NPY* expression across the cell types defined in Figure S6H.

G) UMAP visualization of integrated single-cell transcriptomes from adult δ1/δ2 cells (150 cells each, randomly sampled) and δ-cells from a 13-month-old human infant pancreas.[21] Integration was performed using the fastMNN algorithm. Infant δ-cells segregate into subclusters corresponding to adult δ1 and δ2.

H) Dot plot showing subtype-specific marker gene expression across the two δ-cell populations in infant samples. Although *NPY* is not expressed at this stage, other markers show differential expression, indicating early postnatal δ-cell heterogeneity.

I) UMAP plots showing δ1- and δ2-cell identity based on snATAC-seq (left) and integrated snRNA-seq/snATAC-seq analysis (right) from Augsornworawat et al.[27] Each dot represents a single cell; cell counts are shown in brackets.

J) Heatmap showing promoter accessibility of DEGs between δ1- and δ2-cells (corresponding to Figure 6P).

K) ATAC-seq signal tracks showing chromatin accessibility differences near the *ERBB4* and *NPY* loci in δ1- and δ2-cells.

L) Heatmap of differentially accessible peaks between δ1- and δ2-cells. Statistical analysis was performed using a two-sided logistic regression test.

M) Volcano plot of differential motif accessibility between δ1- and δ2-cells. Top enriched motifs are labeled. Analysis was performed using an unpaired two-sided Wilcoxon rank-sum test ($P \leq 0.01$, fold change ≥ 1.3).

Figure S8

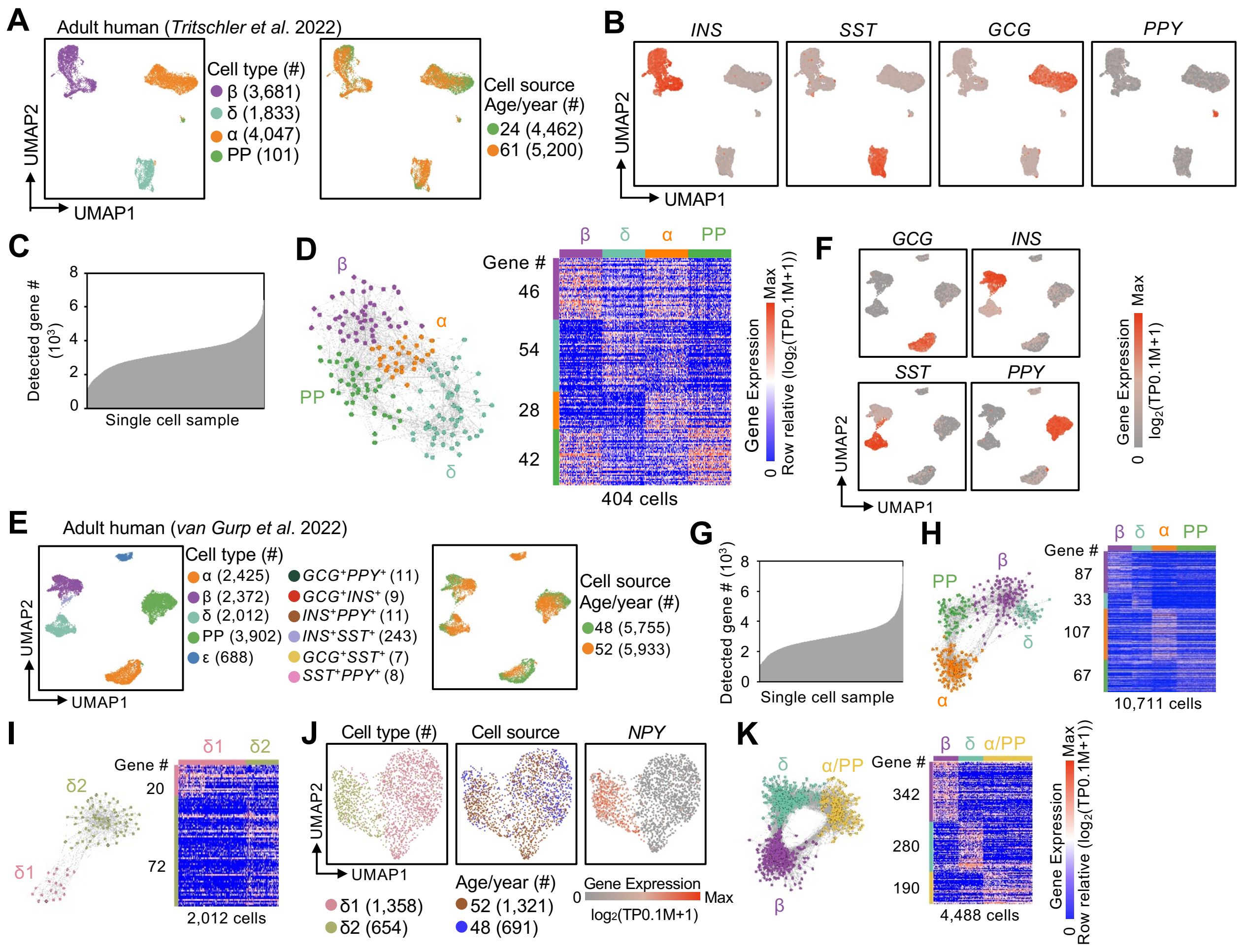
A
Adult human (Tritschler et al. 2022)
Cell type (#)
β (3,681)
δ (1,833)
α (4,047)
PP (101)
Cell source
Age/year (#)
24 (4,462)
61 (5,200)
UMAP2
UMAP1
B
INS
SST
GCG
PPY
C
Detected gene # (10^3)
Single cell sample
D
Gene #
46
54
28
42
404 cells
Gene Expression
Row relative (log2(TP0.1M+1))
E
Adult human (van Gurp et al. 2022)
Cell type (#)
α (2,425)
β (2,372)
δ (2,012)
PP (3,902)
ε (688)
GCG+PPY+ (11)
GCG+INS+ (9)
INS+PPY+ (11)
INS+SST+ (243)
GCG+SST+ (7)
SST+PPY+ (8)
Cell source
Age/year (#)
48 (5,755)
52 (5,933)
F
Gene Expression
log2(TP0.1M+1)
G
Detected gene # (10^3)
Single cell sample
H
Gene #
87
33
107
67
10,711 cells
I
δ1
δ2
Gene #
20
72
2,012 cells
J
Cell type (#)
Cell source
NPY
δ1 (1,358)
δ2 (654)
Age/year (#)
52 (1,321)
48 (691)
Gene Expression
log2(TP0.1M+1)
K
α/PP
Gene #
342
280
190
4,488 cells
Gene Expression
Row relative (log2(TP0.1M+1))

Figure S8. 10x Genomics scRNA-seq analysis and GCN profiling of adult human and mouse pancreatic endocrine cells based on datasets from Tritschler et al.[28] and van Gurp et al.[11b]

A) UMAP plots showing adult human pancreatic endocrine cell types from Tritschler et al.[28] Cell types and sample origins are color-coded.

B) UMAP plots showing expression of representative marker genes for each cell type identified in (A).

C) Statistics of gene counts per cell in thedataset from Tritschler et al.[28]

D) GCN and heatmap analysis showing gene modules differentially expressed among α-, PP-, β-, and δ-cells in adult human islets (Tritschler et al.[28]). Each dot represents a GCN-associated gene; gene counts per cluster are annotated.

E) UMAP plots showing adult human pancreatic endocrine cell types from van Gurp et al.[11b] Cell types and sources are color-coded.

F) UMAP plots displaying expression levels of representative marker genes for each cell type identified in (E).

G) Statistics of the gene counts per cell in the dataset from van Gurp et al.[11b]

H) GCN and heatmap analysis showing differentially expressed gene clusters among α-, PP-, β-, and δ-cells in adult human islets (van Gurp et al.[11b]). Each dot represents a GCN-associated gene; gene counts per cluster are indicated.

I) GCN and heatmap analysis of differentially expressed gene clusters between δ1- and δ2-cells in adult human islets (van Gurp et al.[11b]).

J) UMAP plots of adult human δ-cells showing subpopulation identity (left), sample origin (middle), and *NPY* expression levels (right).

K) GCN and heatmap analysis of differentially expressed gene clusters among the α/PP-cell group, β-cells, and δ-cells in adult mouse islets. Each dot represents a GCN-associated gene; gene counts per cluster are noted. Cell numbers were downsampled to 1,122 per group for cross-species comparison.

Figure S9

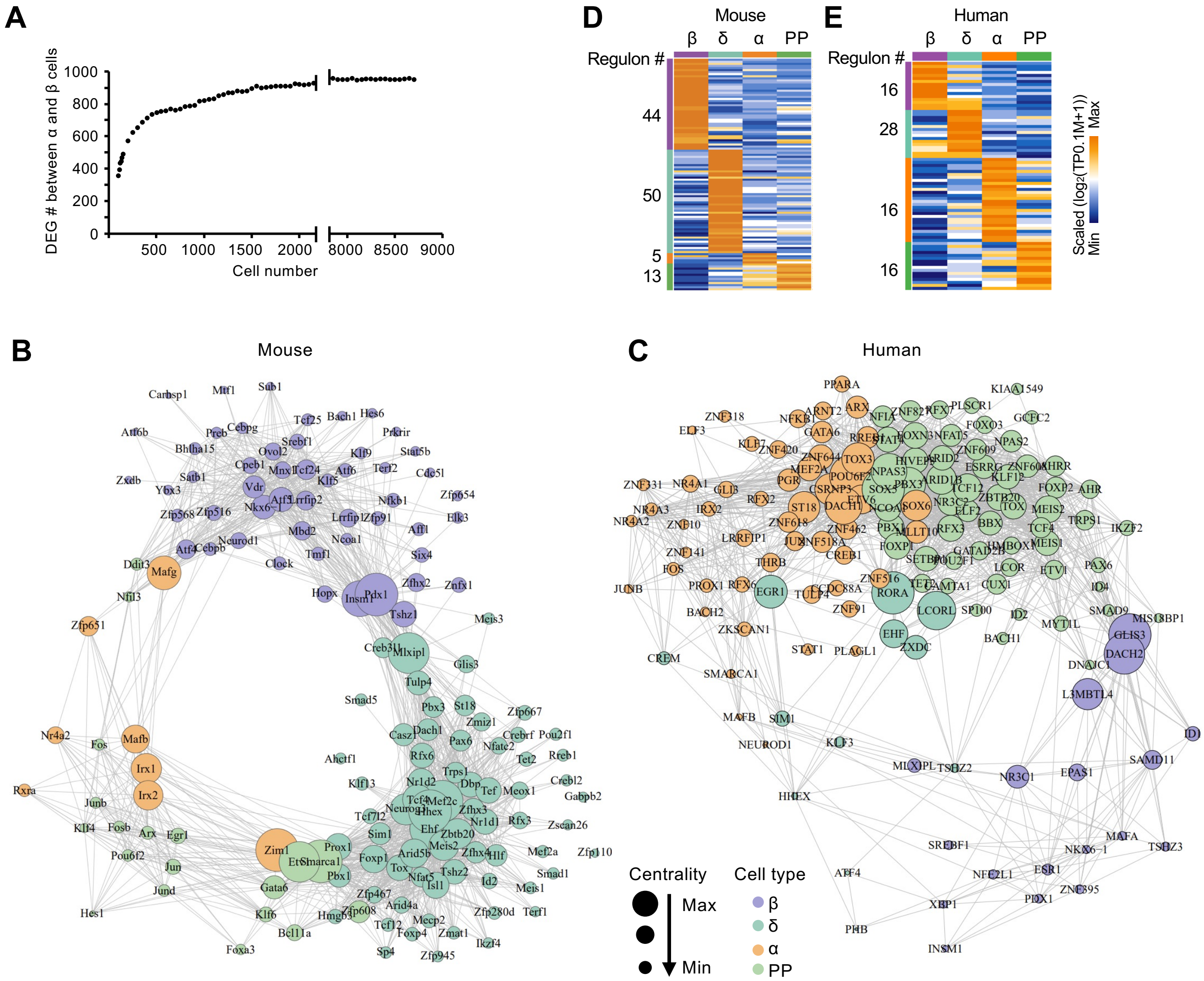
A
DEG # between α and β cells
Cell number
0
200
400
600
800
1000
500
1000
1500
2000
8000
8500
9000
D
Mouse
β
δ
α
PP
Regulon #
44
50
5
13
E
Human
β
δ
α
PP
Regulon #
16
28
16
16
Scaled (log2(TP0.1M+1))
Max
Min
B
Mouse
C
Human
Centrality
Max
Min
Cell type
β
δ
α
PP

Figure S9. Comparative analysis of gene regulatory networks in mouse and human pancreatic endocrine cells.

A) Dot plot showing the number of DEGs between adult human α- and β-cells across varying cell sample sizes. DEGs were identified using the unpaired Wilcoxon rank-sum test with the following thresholds: p-value $\leq 10^{-5}$, $\log_2$(fold change) ≥ 0.5, pct.1 ≥ 0.35, and pct.2 ≤ 0.5.

B, C) GCNs of TFs in mice (B) and humans (C). Each node represents a GCN-associated TF, colored according to cell type specificity. Node size reflects centrality within the network. Edges represent predicted interactions between TFs, with only edges above a weight threshold of 0.1 shown.

D, E) Heatmaps showing expression of target genes within identified regulons across endocrine cell types in mouse (D) and human (E). Each column represents a cell; each row a target gene.

Supplementary Table Legends:

Table S1: Metadata for single cells included in Smart-seq3 scRNA-seq experiments, including sample origin, and cell type annotation.

Table S2: Differentially expressed genes (DEGs) among mouse endocrine cell types across developmental stages.

Table S3: Gene coexpression networks (GCNs) for endocrine cell types in mouse and human islets, including gene module membership and associated metadata.

Table S4: Metadata for cells profiled by 10x Genomics scRNA-seq from P60 mouse pancreatic islets, including cell type classification and sample information.

Table S5: Metadata for human δ-cells profiled by 10x Genomics scRNA-seq, DEGs between δ1 and δ2 subtypes, and Gene Ontology (GO) terms enriched in δ1- and δ2-specific gene clusters.

Table S6: DEGs adult mouse and human endocrine cell types, including transcription factor annotations and fold-change statistics.
